# Correction: Molecular phylogeny and species delimitation of the genus *Tonkinacris *(Orthoptera, Acrididae, Melanoplinae) from China

**DOI:** 10.1371/journal.pone.0256718

**Published:** 2021-08-19

**Authors:** Haojie Wang, Bing Jiang, Jingxiao Gu, Tao Wei, Liliang Lin, Yuan Huang, Dan Liang, Jianhua Huang

In the Funding section, the list of funders is incorrect. The correct Funding statement is as follows: This study is supported by Beijing Forestry University Teaching Reform Project (BJFU2017JY021), the Open foundation for innovation platform of Education Bureau of Hunan Province (18K056) and National Natural Science Foundation of China (No. 31540055, 31260523, 31801993).
