# Supplementary figures and images for: Molecular phylogeny and species delimitation of the genus Tonkinacris (Orthoptera, Acrididae, Melanoplinae) from China
Source: PLoS One. 2021 Apr 13;16(4):e0249431. doi: 10.1371/journal.pone.0249431 (PMC8043412; doi:10.1371/journal.pone.0249431)

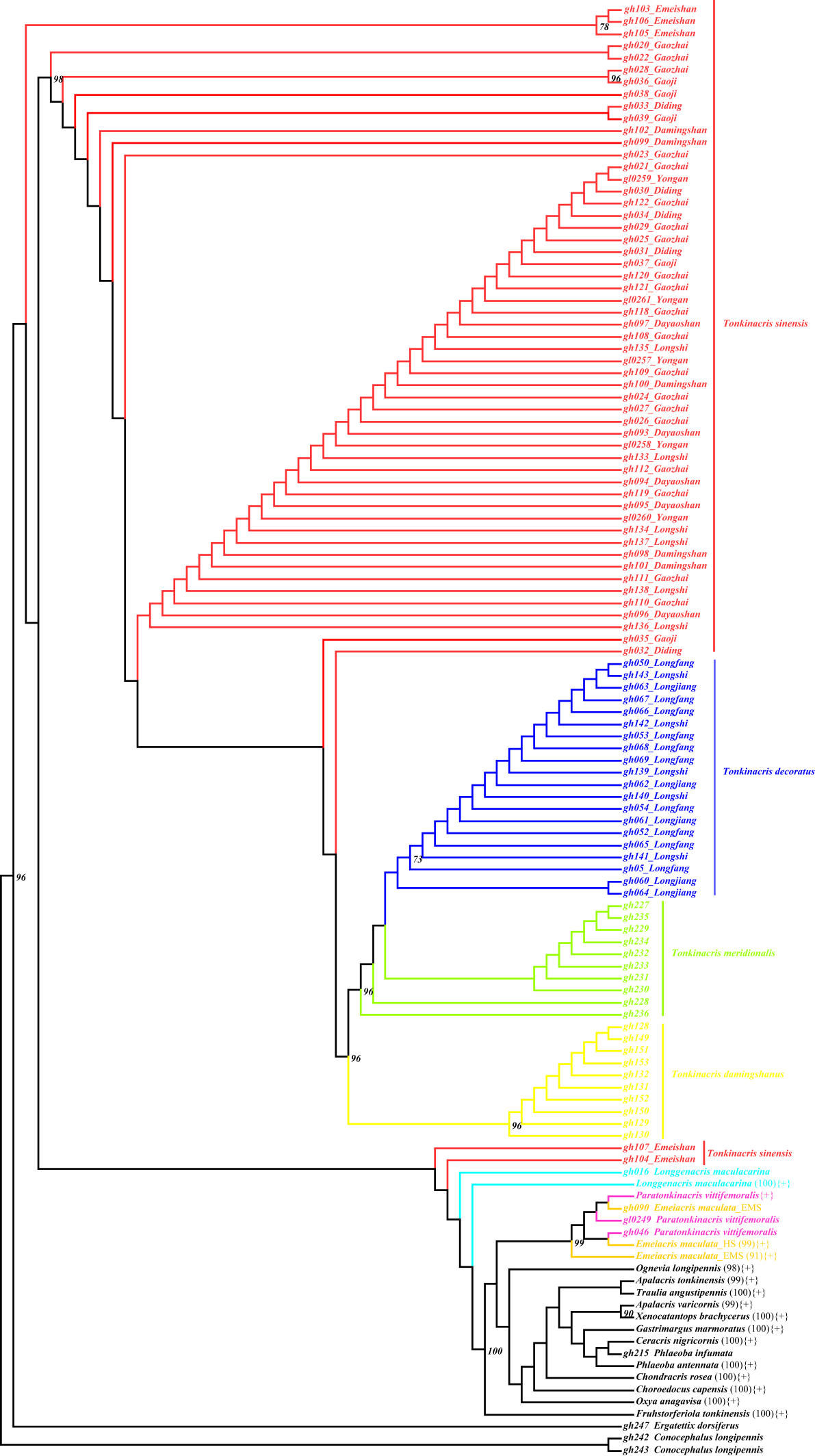


**S1 Fig**. ML tree deduced from ITS1 sequences.

Supplement: S1 Fig — (DOCX) [file pone.0249431.s001.docx]

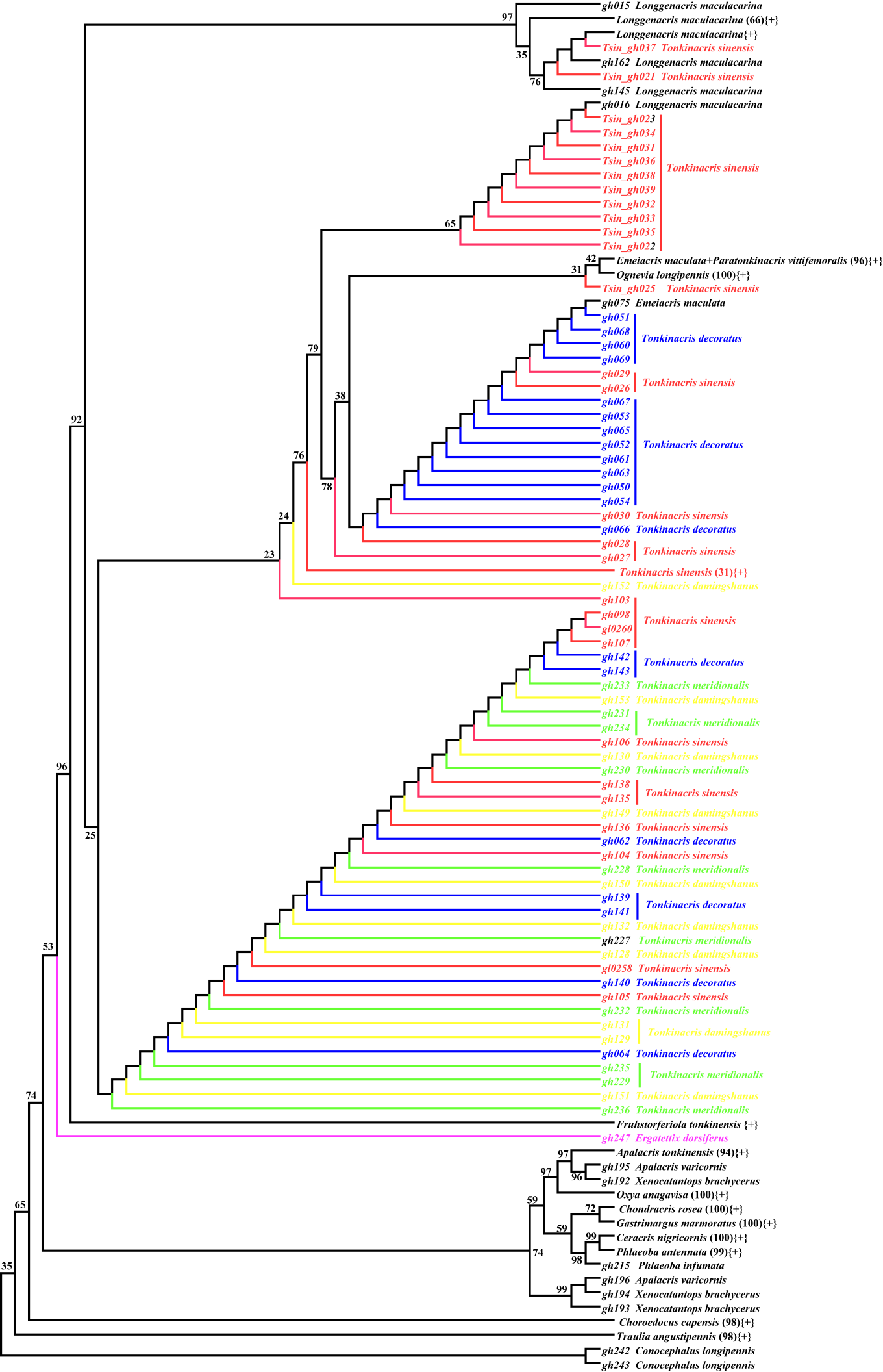


**S2 Fig**. ML tree deduced from ITS2 sequences.

Supplement: S2 Fig — (DOCX) [file pone.0249431.s002.docx]

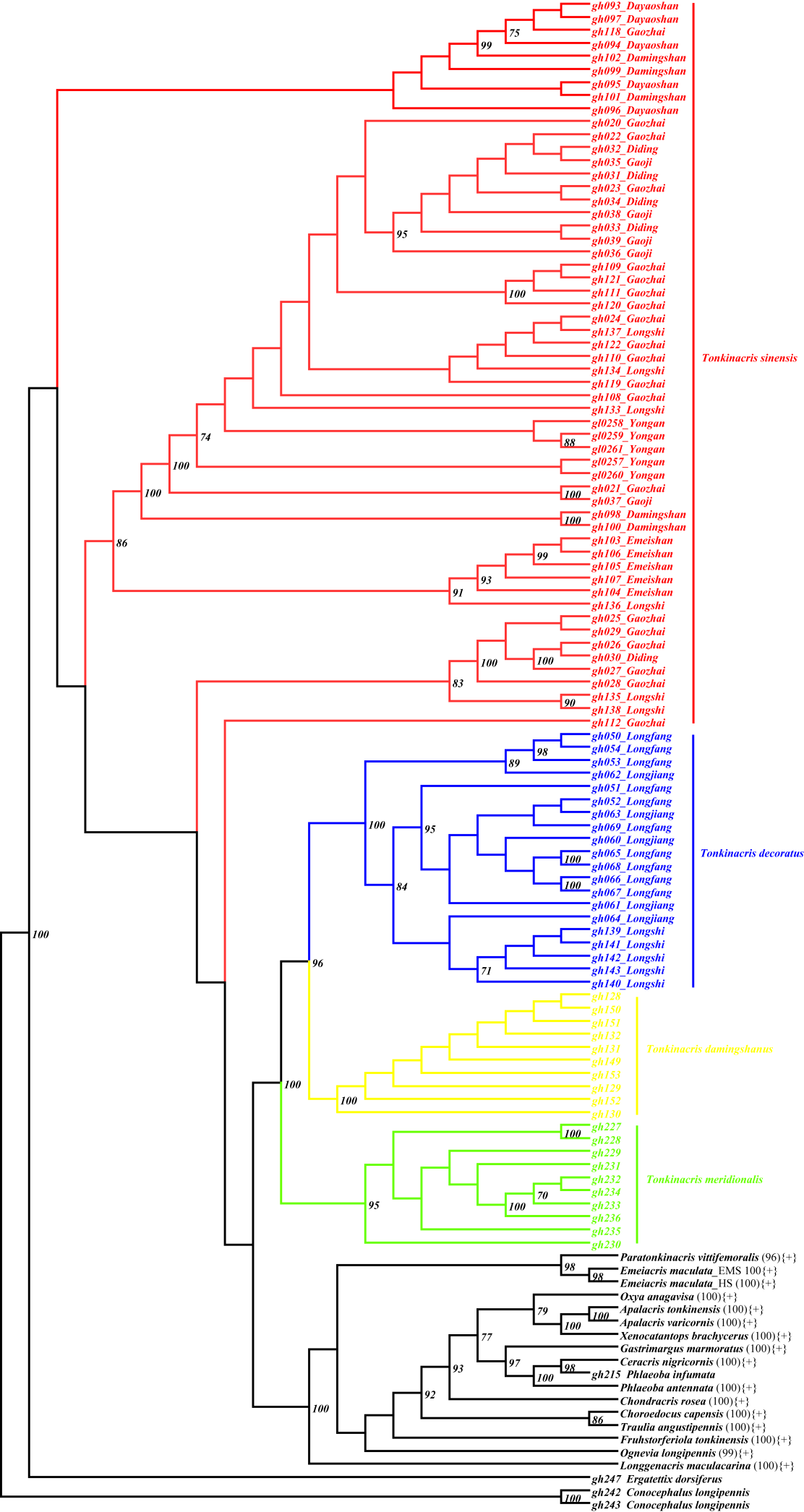


**S3 Fig**. ML tree deduced from combined sequences of mitochondrial *COI*, nuclear ITS1 and ITS2.

Supplement: S3 Fig — (DOCX) [file pone.0249431.s003.docx]

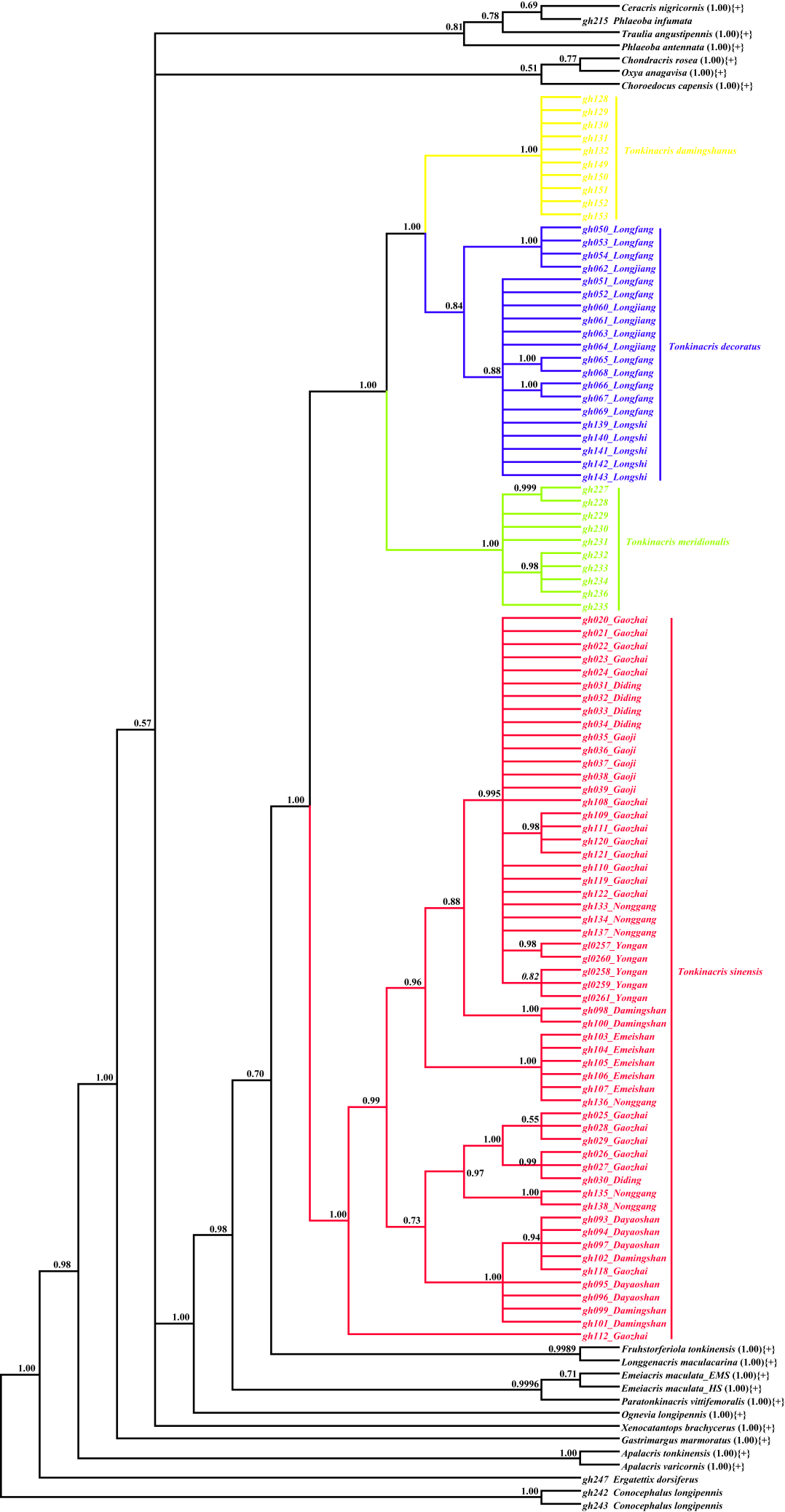


**S4 Fig**. BI tree deduced from COI sequences.

Supplement: S4 Fig — (DOCX) [file pone.0249431.s004.docx]

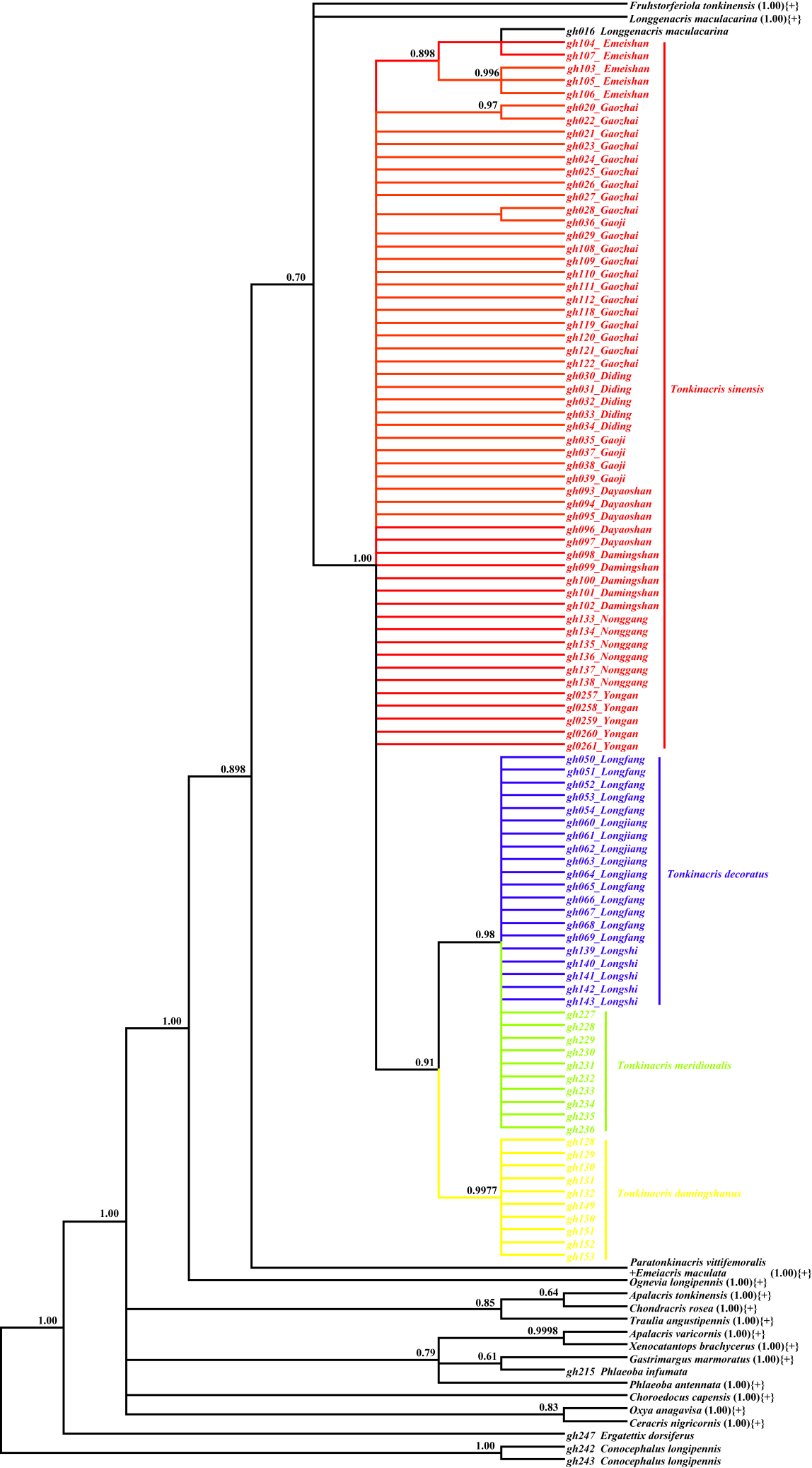


**S5 Fig**. BI tree deduced from ITS1 sequences.

Supplement: S5 Fig — (DOCX) [file pone.0249431.s005.docx]

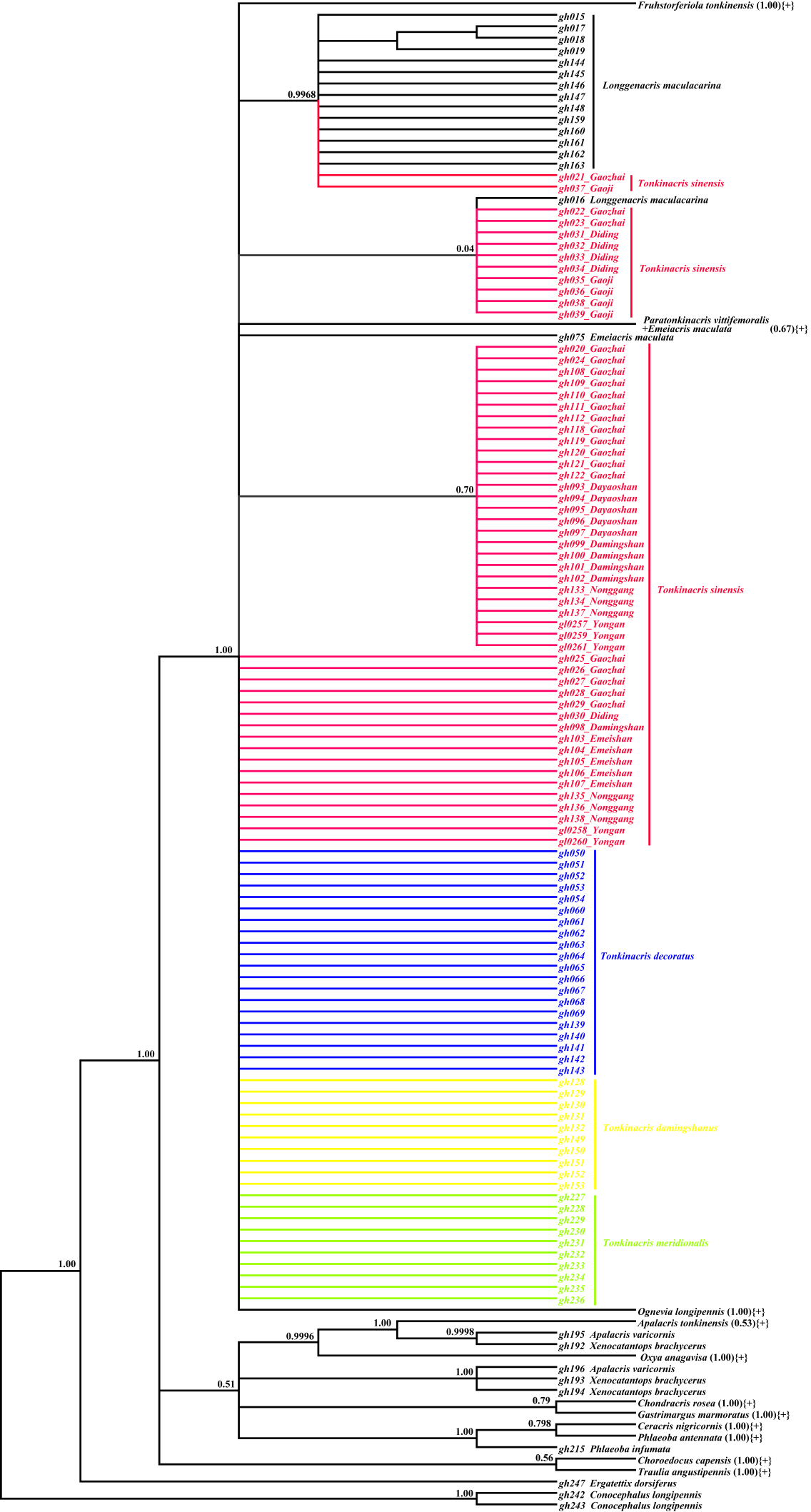


**S6 Fig**. BI tree deduced from ITS2 sequences.

Supplement: S6 Fig — (DOCX) [file pone.0249431.s006.docx]

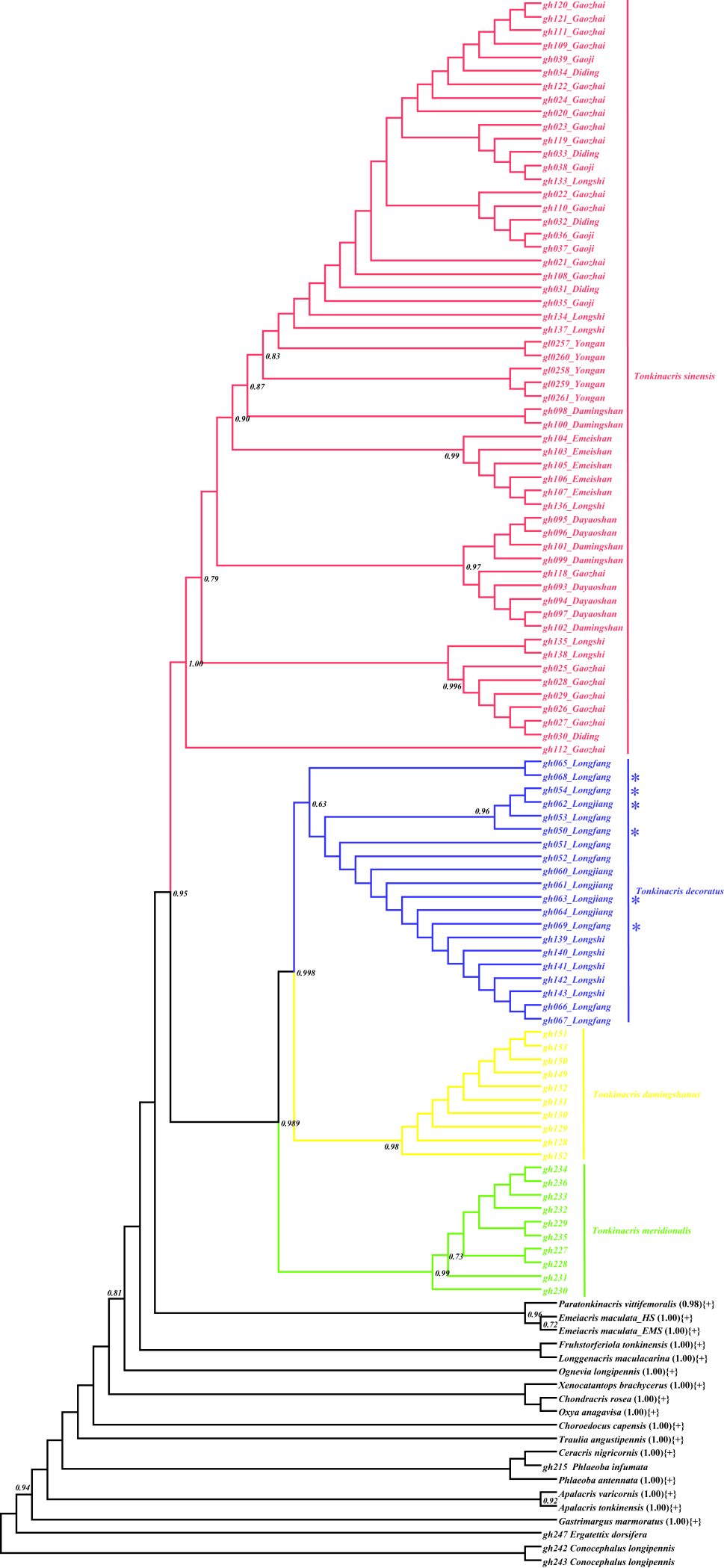


**S7 Fig**. NJ tree deduced from COI sequences.

Supplement: S7 Fig — (DOCX) [file pone.0249431.s007.docx]

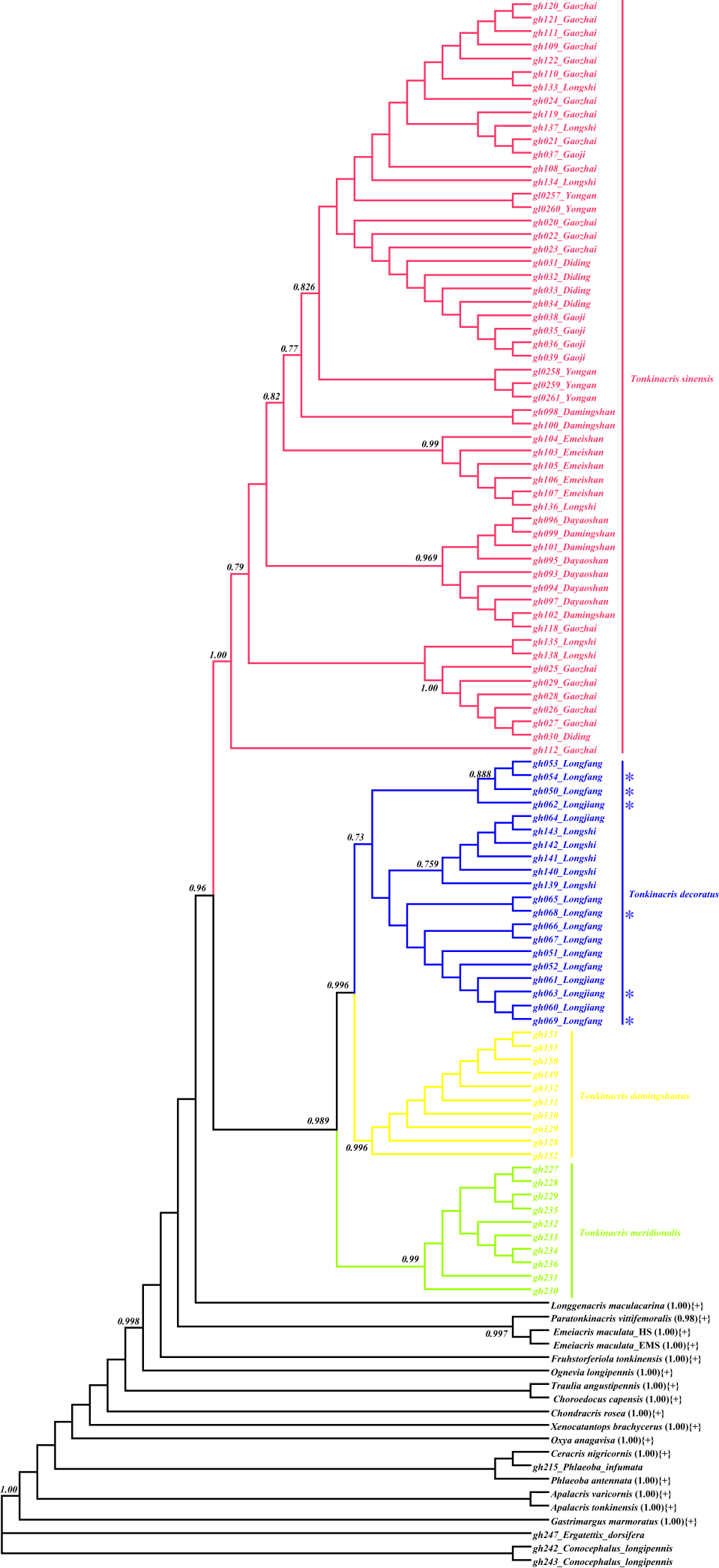


**S8 Fig**. NJ tree deduced from combined sequences of mitochondrial *COI*, nuclear ITS1 and ITS2.

Supplement: S8 Fig — (DOCX) [file pone.0249431.s008.docx]

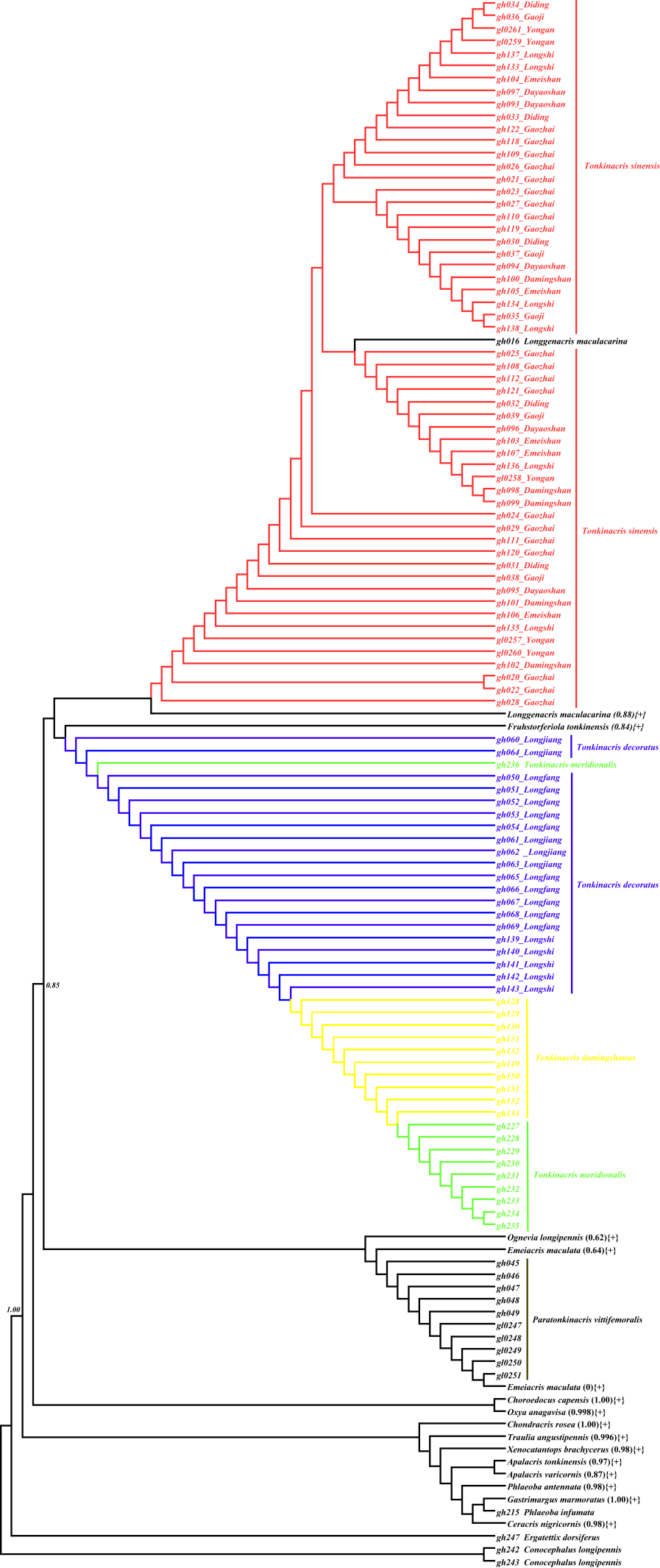


**S9 Fig**. NJ tree deduced from ITS1 sequences.

Supplement: S9 Fig — (DOCX) [file pone.0249431.s009.docx]

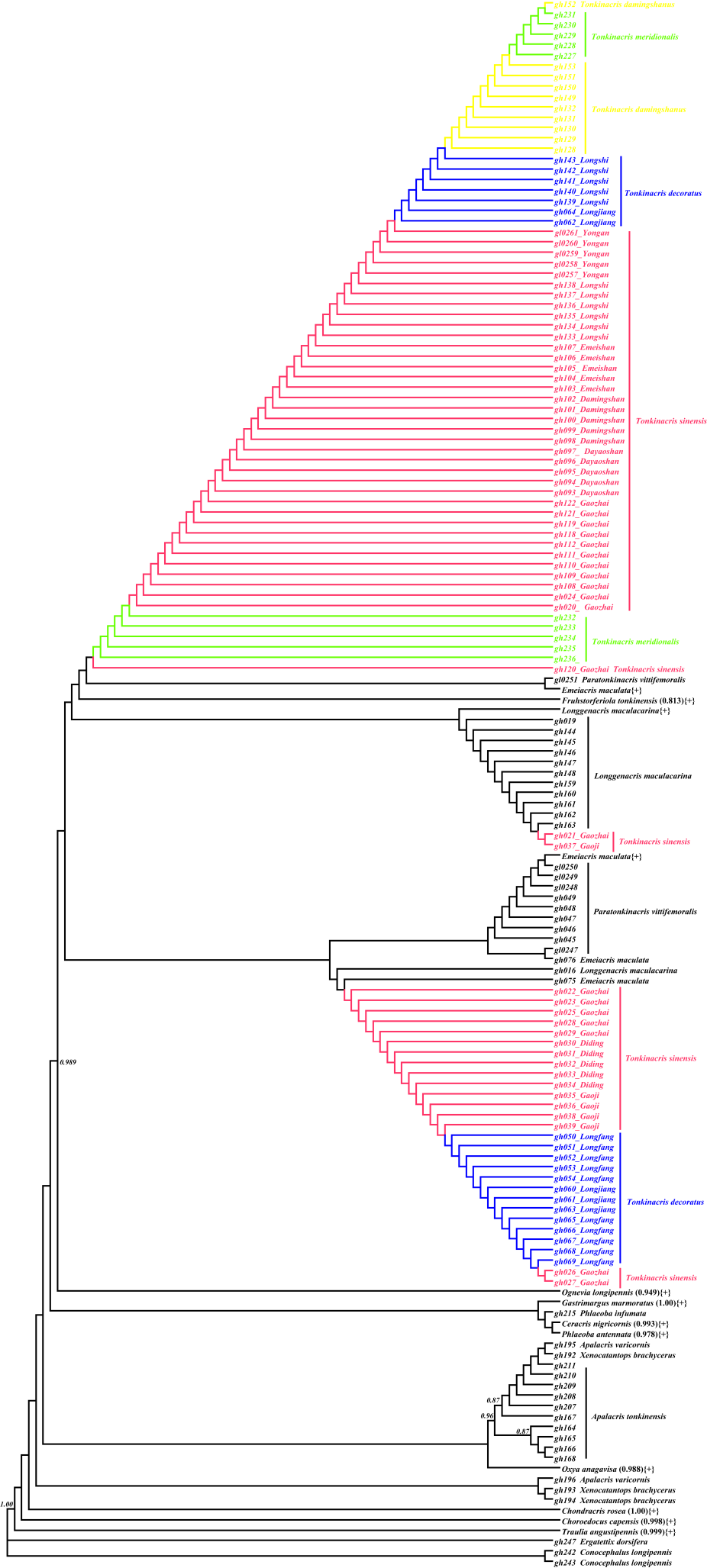


**S10 Fig**. NJ tree deduced from ITS2 sequences.

Supplement: S10 Fig — (DOCX) [file pone.0249431.s010.docx]
