# Supplementary material for: Molecular phylogeny and species delimitation of the genus Tonkinacris (Orthoptera, Acrididae, Melanoplinae) from China
Source: PLoS One. 2021 Apr 13;16(4):e0249431. doi: 10.1371/journal.pone.0249431 (PMC8043412; doi:10.1371/journal.pone.0249431)
Supplement: S1 Table — (DOCX) [file pone.0249431.s011.docx]

**S1 Table.** Molecular data of the genus *Tonkinacris* submitted to NCBI so far.

| Gene | *Tonkinacris sp* | *Tonkinacris sinensis* | *Tonkinacris yaeyamaensis* | *Tonkinacris ruficerus* |
| --- | --- | --- | --- | --- |
| mitogenome |  | NC_032716 (Zhang *et al*., 2017) |  |  |
| *COI* |  | KC139972, KC139973  (Huang *et al*., 2013) | KC261367 (Bugrov *et al*., 2012),  KX440512  (Grzywacz & Tatsuta, 2017) | KX440510, KX440511  (Grzywacz & Tatsuta, 2017) |
| *COII* | KF960028  (Chintauan-Marquier *et al*., 2014) | DQ118036 (Ma & Huang, 2006) |  |  |
| *Cytb* |  | DQ366752 (Lu *et al*., 2010)  DQ365910 (Huo *et al*., 2007) |  |  |
| *NDH2* |  | DQ092565 (Chen & Huang, 2005) |  |  |
| *16S* | KF729506  (Chintauan-Marquier *et al*., 2014) | DQ366812 (Lu *et al*., 2010),  AY566261 (Liu *et al*., 2005) |  |  |
| *12S* | KF705061  (Chintauan-Marquier *et al*., 2014) | AY247186  (Jiang & Zhang, 2003; unpulished) |  |  |
| *18S*-ITS1-  *5.8S*-ITS2 | KF924587  (Chintauan-Marquier *et al*., 2014) | MH934174, MH934175  (Gu *et al*., 2020), |  |  |
| *28S* |  | DQ366781 (Lu *et al*., 2010) |  |  |

# 
