# Supplementary material for: Molecular phylogeny and species delimitation of the genus Tonkinacris (Orthoptera, Acrididae, Melanoplinae) from China
Source: PLoS One. 2021 Apr 13;16(4):e0249431. doi: 10.1371/journal.pone.0249431 (PMC8043412; doi:10.1371/journal.pone.0249431)
Supplement: S2 Table — (DOCX) [file pone.0249431.s012.docx]

**S2 Table.** Materials involved in this study.

| Species | Voucher number | Collecting data |
| --- | --- | --- |
| **Caelifera, Acridoidea, Acrididae**  **Melanoplinae** |  |  |
| *Emeiacris maculata* | gh075-079, 088-092 | Hongchunping, Emeishan, Leshan County, Sichuan, China; 27 July 2011; Ruigang Yang leg. |
|  | gl0241-0246 | Hengshan, Hunan, China; 29 August 2008; Jianhua Huang leg. |
| *Fruhstorferiola tonkinensis* | gl0089-0094 | Yong'an, Xing'an, Guangxi, China; 2 July 2006; Jianhua Huang leg. |
| *Longgenacris maculacarina* | gh015-019 | Longjiang, Nonggang, Longzhou County, Guangxi, China; 27 July 2012; Tao Wei leg. |
|  | gh144-148 | Longfang, Nonggang, Longzhou County, Guangxi, China; 25 August 2012; Tao Wei leg. |
|  | gh159-163 | Longshi, Nonggang, Longzhou County, Guangxi, China; 28 July 2012; Tao Wei leg. |
| *Ognevia longipennis* | gl0252-0256 | Yangjiaping, Zhuolu, Hebei, China; 20 August 2005; Yuan Huang leg. |
| *Paratonkinacris vittifemoralis* | gh045-049, gl0247-0251 | Gaozhai, Xing'an, Guangxi, China; 8 July 2009; Jianhua Huang leg. |
| *Tonkinacris damingshanus* | gh128-132 | Yuanshisenlin, Damingshan, Wuming County, Guangxi, China; 29 July 2012; Ruigang Yang leg. |
|  | gh149-153 | Yuanshisenlin, Damingshan, Wuming County, Guangxi, China; 8 August 2012; Ruigang Yang leg. |
| *Tonkinacris decoratus* | gh050-054, 065-069 | Longfang, Nonggang, Longzhou County, Guangxi, China; 25 August 2012; Tao Wei leg. |
|  | gh060-064 | Longjiang, Nonggang, Longzhou County, Guangxi, China; 29 July 2012; Tao Wei leg. |
|  | gh139-143 | Longshi, Nonggang, Longzhou County, Guangxi, China; 28 July 2012; Tao Wei leg. |
| *Tonkinacris meridionalis* | gh227-236 | Longrui, Longzhou County, Guangxi, China; 25 July 2013; Taowei leg. |
| *Tonkinacris sinensis* | gh020-029, 118-122 | Gaozhai, Xing'an County, Guangxi, China; 8 July 2009; Jianhua Huang leg. |
|  | gh108-112 | Gaozhai, Xing'an County, Guangxi, China; 18 September 2011; Chunwen Lu leg. |
|  | gh030-034 | Diding, Jingxi County, Guangxi, China; 8 August 2010; Jianhua Huang leg. |
|  | gh035-039 | Gaoji Town, Sanjiang County, Guangxi, China; 11 July 2009; Jianhua Huang leg. |
|  | gh093-097 | Qigongli, Dayaoshan, Jinxiu County, Guangxi, China; 15 August 2011; Bingchui Su leg. |
|  | gh098-102 | Xiashuiyuan Village, Damingshan, Shanglin County, Guangxi, China; 15 September 2011; Ruigang Yang leg. |
|  | gh103-107 | Fuhusi, Emeishan, Leshan County, Sichuan, China; 20 July 2011; Ruigang Yang leg. |
|  | gh133-138 | Longshi, Nonggang, Longzhou County, Guangxi, China; 28 July 2012; Tao Wei leg. |
|  | gl0257-0261 | Yong'an, Xing'an County, Guangxi, China; 2 July 2006; Jianhua Huang leg. |
| **Coptacrinae** |  |  |
| *Apalacris tonkinensis* | gh164-168 | Longjiang, Nonggang, Longzhou County, Guangxi, China; 27 August 2012; Tao Wei leg. |
|  | gh207-211 | Longshi, Nonggang, Longzhou County, Guangxi, China; 29 July 2012; Tao Wei leg. |
| *Apalacris varicornis* | gh195-196 | Sanlian, Nonggang, Longzhou County, Guangxi, China; 1 September 2012; Tao Wei leg. |
| **Cyrtacanthacridinae** |  |  |
| *Chondracris rosea* | gh174-178 | Longjiang, Nonggang, Longzhou County, Guangxi, China; 28 July 2012; Tao Wei leg. |
| **Eyprepocnemidinae** |  |  |
| *Choroedocus capensis* | gh170-173 | Longjiang, Nonggang, Longzhou County, Guangxi, China; 28 July 2012; Tao Wei leg. |
|  | gh184-191 | Sanlidian, Guilin City, Guangxi, China; 15 October 15 October 2009; Jianhua Huang leg. |
| **Catantopinae** |  |  |
| *Xenocatantops brachycerus* | gh192-194 | Sanlian, Nonggang, Longzhou County, Guangxi, China; 1 September 2012; Tao Wei leg. |
| *Traulia angustipennis* | gh221-226 | Sanlian, Nonggang, Longzhou County, Guangxi, China; 1 September 2012; Tao Wei leg. |
| **Oxyinae** |  |  |
| *Oxya anagavisa* | gh070-074 | Lanshengzhilv, Damingshan, Wuming County, Guangxi, China; 8 September 2011; Ruigang Yang leg. |
|  | gh179-180 | Longjiang, Nonggang, Longzhou County, Guangxi, China; 27 July 2012; Tao Wei leg. |
|  | gh202-206 | Longgu, Nonggang, Longzhou County, Guangxi, China; 18 August 2012; Tao Wei leg. |
| **Oedipodinae** |  |  |
| *Gastrimargus marmoratus* | gh181-183 | Longjiang, Nonggang, Longzhou County, Guangxi, China; 27 July 2012; Tao Wei leg. |
| *Ceracris nigricornis* | gh197-201 | Sanlian, Nonggang, Longzhou County, Guangxi, China; 1 September 2012; Tao Wei leg. |
| **Acridinae** |  |  |
| *Phlaeoba antennata* | gh212-214 | Longshi, Nonggang, Longzhou County, Guangxi, China; 29 July 2012; Tao Wei leg. |
|  | gh216-220 | Nonggang, Longzhou County, Guangxi, China; 26 July 2012; Tao Wei leg. |
| *Phlaeoba infumata* | gh215 | Longshi, Nonggang, Longzhou County, Guangxi, China; 29 July 2012; Tao Wei leg. |
| **Tetrigoidea, Tetrigidae** |  |  |
| *Ergatettix dorsiferus* | gh247 | Fangchenggang, Guangxi, China; 18 December 2013; Chunwen Lu leg. |
| **Ensifera, Tettigoniidae** |  |  |
| *Conocephalus longipennis* | gh242-243 | Lengjia Village, Guilin City, Guangxi, China; 23 October 2013; Tao Wei leg. |
