# Supplementary material for: Molecular phylogeny and species delimitation of the genus Tonkinacris (Orthoptera, Acrididae, Melanoplinae) from China
Source: PLoS One. 2021 Apr 13;16(4):e0249431. doi: 10.1371/journal.pone.0249431 (PMC8043412; doi:10.1371/journal.pone.0249431)
Supplement: S3 Table — (DOCX) [file pone.0249431.s013.docx]

**S3 Table.** Mapping table between GenBank accession numbers and voucher numbers.

| Voucher number | GenBank accession number | | | Voucher number | GenBank accession number | | |
| --- | --- | --- | --- | --- | --- | --- | --- |
|  | COI | ITS1 | ITS2 |  | COI | ITS1 | ITS2 |
| gh015 | MH934117 | MH934156 | MH934156 | gh152 | MW056473* | MW054585* | MW054585* |
| gh016 | MH934117 | MH934157 | MH934157 | gh153 | MW053522* | MW054585* | MW054585* |
| gh017 | MH934117 | MH934158 | MH934158 | gh159 | MH934117 | MH934160 | MH934160 |
| gh018 | MH934117 | MH934158 | MH934158 | gh160 | MH934118 | MH934160 | MH934160 |
| gh019 | MH934117 | MH934159 | MH934159 | gh161 | MH934119 | MH934160 | MH934160 |
| gh020 | MW053510* | MW054567* | MW054567* | gh162 | MH934117 | MH934160 | MH934160 |
| gh021 | MW053510* | MW054567* | MW054567* | gh163 | MH934120 | MH934160 | MH934160 |
| gh022 | MW053510* | MW054567* | MW054567* | gh164 | MH934125 | MH934179 | MH934179 |
| gh023 | MW053510* | MW054567* | MW054567* | gh165 | MH934125 | MH934180 | MH934180 |
| gh024 | MW053510* | MW054567* | MW054567* | gh166 | MH934126 | MH934180 | MH934180 |
| gh025 | MW053511* | MW054567* | MW054567* | gh167 | MH934125 | MH934181 | MH934181 |
| gh026 | MW056459* | MW054567* | MW054567* | gh168 | MH934125 | MH934180 | MH934180 |
| gh027 | MW056459* | MW054567* | MW054567* | gh170 | MW053524* | MW054590* | MW054590* |
| gh028 | MW053511* | MW055691* | MW055691* | gh171 | MW053524* | MW054590* | MW054590* |
| gh029 | MW053511* | MW054567* | MW054567* | gh172 | MW053525* | MW054591* | MW054591* |
| gh030 | MW056459* | MW054567* | MW054567* | gh173 | MW053525* | MW054590* | MW054590* |
| gh031 | MW053510* | MW054567* | MW054567* | gh174 | MW053526* | MW054592* | MW054592* |
| gh032 | MW053510* | MW055692* | MW055692* | gh175 | MW053527* | MW054593* | MW054593* |
| gh033 | MW053510* | MW055693* | MW055693* | gh176 | MW053528* | MW054594* | MW054594* |
| gh034 | MW053510* | MW054567* | MW054567* | gh177 | MW053529* | MW054592* | MW054592* |
| gh035 | MW053510* | MW055694* | MW055694* | gh178 | MW053530* | MW054596* | MW054596* |
| gh036 | MW053510* | MW055695* | MW055695* | gh179 | MW053537* | MW054576* | MW054576* |
| gh037 | MW056460* | MW054567* | MW054567* | gh180 | MW053537* | MW054576* | MW054576* |
| gh038 | MW053510* | MW055693* | MW055693* | gh181 | MW053530* | MW054598* | MW054598* |
| gh039 | MW053510* | MW055693* | MW055693* | gh182 | MW053531* | MW054599* | MW054599* |
| gh045 | MH934121 | MH934161 | MH934161 | gh183 | MW053532* | MW054600* | MW054600* |
| gh046 | MH934121 | MH934161 | MH934161 | gh184 | MW053533* | MW054590* | MW054590* |
| gh047 | MH934121 | MH934161 | MH934161 | gh185 | MW053533* | MW054590* | MW054590* |
| gh048 | MH934121 | MH934161 | MH934161 | gh186 | MW053533* | MW055699* | MW055699* |
| gh049 | MH934122 | MH934161 | MH934161 | gh187 | MW053533* | MW054590* | MW054590* |
| gh050 | MW053516* | MW054571* | MW054571* | gh188 | MW053533* | MW055699* | MW055699* |
| gh051 | MW056461* | MW054571* | MW054571* | gh189 | MW053533* | MW054590* | MW054590* |
| gh052 | MW053517* | MW054571* | MW054571* | gh190 | MW053533* | MW055699* | MW055699* |
| gh053 | MW053516* | MW054571* | MW054571* | gh191 | MW053533* | MW055699* | MW055699* |
| gh054 | MW053516* | MW054571* | MW054571* | gh192 | MW053534* | MW054602* | MW054602* |
| gh060 | MW053517* | MW055696* | MW055696* | gh193 | MW056474* | MW054602* | MW054602* |
| gh061 | MW053517* | MW054571* | MW054571* | gh194 | MW056475* | MW054602* | MW054602* |
| gh062 | MW053516* | MW054571* | MW054571* | gh195 | MW056476* | MW054605* | MW054605* |
| gh063 | MW053517* | MW054571* | MW054571* | gh196 | MW056477* | MW054605* | MW054605* |
| gh064 | MW053517* | MW054571* | MW054571* | gh197 | MW053536* | MW054607* | MW054607* |
| gh065 | MW056462* | MW054571* | MW054571* | gh198 | MW056478* | MW054607* | MW054607* |
| gh066 | MW056463* | MW054571* | MW054571* | gh199 | MW056479* | MW054607* | MW054607* |
| gh067 | MW056463* | MW054571* | MW054571* | gh200 | MW056480* | MW054607* | MW054607* |
| gh068 | MW056462* | MW054571* | MW054571* | gh201 | MW056478* | MW054607* | MW054607* |
| gh069 | MW053517* | MW054571* | MW054571* | gh202 | MW053537* | MW054576* | MW054576* |
| gh070 | MW053518* | MW054576* | MW054576* | gh203 | MW053537* | MW055700* | MW055700* |
| gh071 | MW053518* | MW054576* | MW054576* | gh204 | MW053537* | MW054576* | MW054576* |
| gh072 | MW053518* | MW055697* | MW055697* | gh205 | MW053537* | MW054576* | MW054576* |
| gh073 | MW053518* | MW055697* | MW055697* | gh206 | MW053537* | MW055697* | MW055697* |
| gh074 | MW053518* | MW054576* | MW054576* | gh207 | MH934125 | MH934182 | MH934182 |
| gh075 | MH934123 | MH934164 | MH934164 | gh208 | MH934126 | MH934183 | MH934183 |
| gh076 | MH934123 | MH934165 | MH934165 | gh209 | MH934126 | MH934181 | MH934181 |
| gh077 | MH934123 | MH934166 | MH934166 | gh210 | MH934125 | MH934181 | MH934181 |
| gh078 | MH934123 | MH934167 | MH934167 | gh211 | MH934125 | MH934184 | MH934184 |
| gh079 | MH934123 | MH934168 | MH934168 | gh212 | MW053538* | MW054611* | MW054611* |
| gh088 | MH934123 | MH934169 | MH934169 | gh213 | MW053539* | MW054611* | MW054611* |
| gh089 | MH934123 | MH934170 | MH934170 | gh214 | MW053539* | MW055701* | MW055701* |
| gh090 | MH934123 | MH934161 | MH934161 | gh215 | MW053540* | MW054613* | MW054613* |
| gh091 | MH934124 | MH934171 | MH934171 | gh216 | MW053541* | MW054614* | MW054614* |
| gh092 | MH934123 | MH934170 | MH934170 | gh217 | MW056481* | MW054611* | MW054611* |
| gh093 | MW056464* | MW054567* | MW054567* | gh218 | MW056482* | MW054611* | MW054611* |
| gh094 | MW056464* | MW054567* | MW054567* | gh219 | MW056482* | MW054611* | MW054611* |
| gh095 | MW056465* | MW054567* | MW054567* | gh220 | MW056482* | MW054614* | MW054614* |
| gh096 | MW056466* | MW054567* | MW054567* | gh221 | MW053542* | MW054617* | MW054617* |
| gh097 | MW056464* | MW054567* | MW054567* | gh222 | MW056483* | MW054617* | MW054617* |
| gh098 | MW056467* | MW054567* | MW054567* | gh223 | MW053543* | MW054619* | MW054619* |
| gh099 | MW056465* | MW054567* | MW054567* | gh224 | MW056484* | MW054617* | MW054617* |
| gh100 | MW056467* | MW054567* | MW054567* | gh225 | MW056484* | MW054617* | MW054617* |
| gh101 | MW056465* | MW054567* | MW054567* | gh226 | MW056483* | MW054617* | MW054617* |
| gh102 | MW056464* | MW054567* | MW054567* | gh227 | MW053544* | MW054626* | MW054626* |
| gh103 | MW053520* | MW054581* | MW054581* | gh228 | MW053544* | MW054626* | MW054626* |
| gh104 | MW053521* | MW054582* | MW054582* | gh229 | MW056485* | MW054626* | MW054626* |
| gh105 | MW053520* | MW054581* | MW054581* | gh230 | MW056486* | MW054626* | MW054626* |
| gh106 | MW053520* | MW054581* | MW054581* | gh231 | MW056487* | MW054626* | MW054626* |
| gh107 | MW053520* | MW054582* | MW054582* | gh232 | MW056488* | MW054626* | MW054626* |
| gh108 | MW053510* | MW054567* | MW054567* | gh233 | MW056488* | MW054626* | MW054626* |
| gh109 | MW056468* | MW054567* | MW054567* | gh234 | MW056488* | MW054626* | MW054626* |
| gh110 | MW053510* | MW054567* | MW054567* | gh235 | MW056489* | MW054626* | MW054626* |
| gh111 | MW056468* | MW054567* | MW054567* | gh236 | MW056488* | MW054626* | MW054626* |
| gh112 | MW056469* | MW054567* | MW054567* | gh242 | MH934128 | MH934186 | MH934186 |
| gh118 | MW056464* | MW054567* | MW054567* | gh243 | MH934129 | MH934186 | MH934186 |
| gh119 | MW053510* | MW054567* | MW054567* | gh247 | MH934127 | MH934185 | MH934185 |
| gh120 | MW056468* | MW054567* | MW054567* | gl0089 | KC139886 | MH934141 | MH934141 |
| gh121 | MW056468* | MW054567* | MW054567* | gl0090 | KC139887 | MH934140 | MH934140 |
| gh122 | MW053510* | MW054567* | MW054567* | gl0091 | KC139888 | MH934140 | MH934140 |
| gh128 | MW053522* | MW054585* | MW054585* | gl0092 | KC139889 | MH934140 | MH934140 |
| gh129 | MW053522* | MW054585* | MW054585* | gl0093 | KC139890 | MH934140 | MH934140 |
| gh130 | MW053522* | MW055698* | MW055698* | gl0094 | KC139890 | MH934140 | MH934140 |
| gh131 | MW053522* | MW054585* | MW054585* | gl0241 | KC139851 | MH934172 | MH934172 |
| gh132 | MW053522* | MW054585* | MW054585* | gl0242 | KC139852 | MH934172 | MH934172 |
| gh133 | MW056470* | MW054567* | MW054567* | gl0243 | KC139853 | MH934172 | MH934172 |
| gh134 | MW053510* | MW054568* | MW054568* | gl0244 | KC139854 | MH934172 | MH934172 |
| gh135 | MW056471* | MW054569* | MW054569* | gl0245 | KC139855 | MH934173 | MH934173 |
| gh136 | MW053520* | MW054570* | MW054570* | gl0246 | KC139856 | MH934173 | MH934173 |
| gh137 | MW053510* | MW054571* | MW054571* | gl0247 | KC139857 | MH934162 | MH934162 |
| gh138 | MW056472* | MW054572* | MW054572* | gl0248 | KC139858 | MH934161 | MH934161 |
| gh139 | MW053517* | MW054571* | MW054571* | gl0249 | KC139858 | MH934161 | MH934161 |
| gh140 | MW053517* | MW054571* | MW054571* | gl0250 | KC139859 | MH934161 | MH934161 |
| gh141 | MW053517* | MW054571* | MW054571* | gl0251 | KC139860 | MH934163 | MH934163 |
| gh142 | MW053517* | MW054571* | MW054571* | gl0252 | KC139896 | MH934176 | MH934176 |
| gh143 | MW053517* | MW054571* | MW054571* | gl0253 | KC139897 | MH934177 | MH934177 |
| gh144 | MH934118 | MH934160 | MH934160 | gl0254 | KC139896 | MH934177 | MH934177 |
| gh145 | MH934117 | MH934160 | MH934160 | gl0255 | KC139896 | MH934178 | MH934178 |
| gh146 | MH934118 | MH934160 | MH934160 | gl0256 | KC139898 | MH934178 | MH934178 |
| gh147 | MH934117 | MH934160 | MH934160 | gl0257 | KC139972 | MH934174 | MH934174 |
| gh148 | MH934117 | MH934160 | MH934160 | gl0258 | KC139973 | MH934175 | MH934175 |
| gh149 | MW053522* | MW054585* | MW054585* | gl0259 | KC139973 | MH934174 | MH934174 |
| gh150 | MW053522* | MW054585* | MW054585* | gl0260 | KC139972 | MH934175 | MH934175 |
| gh151 | MW053522* | MW054585* | MW054585* | gl0261 | KC139973 | MH934174 | MH934174 |

Note. The asterisk symbol "*" indicates the sequence generated in the present study.
