# Supplementary material for: Molecular phylogeny and species delimitation of the genus Tonkinacris (Orthoptera, Acrididae, Melanoplinae) from China
Source: PLoS One. 2021 Apr 13;16(4):e0249431. doi: 10.1371/journal.pone.0249431 (PMC8043412; doi:10.1371/journal.pone.0249431)
Supplement: S4 Table — (DOCX) [file pone.0249431.s014.docx]

**S4 Table.** Intraspecific variations calculated from different datasets.

| Species/populations | Intraspecific variations (mean/pair distance) | | |
| --- | --- | --- | --- |
|  | *COI* | ITS1 | ITS2 |
| *Fruhstorferiola tonkiensis* | 0.49% (0–0.77%) | 0. 05% (0–0.16%) | 0 (0) |
| *Longgenaris maculacarina* | 0.17% (0–0.61%) | 0. 24% (0–**1.77**%) | 0. 39% (0–**1.70**%) |
| *Paratonkinacris vittifemoralis* | 0.46% (0–1.38%) | 0 (0) | 0. 21% (0–0.84%) |
| *Emeiacris maculata* | 2.28% (0–**4.73**%) | 0. 33% (0–0.82%) | 0. 40% (0–1.41%) |
| *E. maculata*_Emeishan | 0.03% (0–0.15%) | 0.26% (0–**0.66**%) | 0. 49% (0-**1.41**%) |
| *E. maculata*_hengshan | 0.36% (0.15–0.61%) | 0 (0) | 0.30% (0-0.56%) |
| *Tonkinacris sinensis* | 1.10% (0–**3.29**%) | 0. 12% (0–1.29%) | 0. 44% (0–1.70%) |
| *T. sinensis*_Diding | 0. 74% (0–1.86%) | 0 (0) | 0.11% (0-0.28%) |
| *T. sinensis*_Gaoji | 0. 06% (0–0.15%) | 0.45% (0–**1.13**%) | 0.68% (0-**1.70**%) |
| *T. sinensis*_Dayaoshan | 0. 15% (0–0.30%) | 0 (0) | 0 (0) |
| *T. sinensis*_Damingshan | 0.1.08% (0–1.86%) | 0.19% (0–0.48%) | 0.11% (0-0.28%) |
| *T. sinensis*_Emeishan | 0. 06% (0–0.15%) | 0.10% (0–0.16%) | 0 (0) |
| *T. sinensis*_Nonggang | 1.08% (0–1.70%) | 0 (0) | 0.17% (0-0.28%) |
| *T. sinensis*_Gaozhai | 1.10% (0–**3.13**%) | 0.06% (0–0.48%) | 0.53% (0–1.70%) |
| *T. sinensis*_Yongan | 0. 18% (0–0.30%) | 0 (0) | 0.17% (0-0.28%) |
| *Tonkinacris decoratus* | 0. 31% (0–0.92%) | 0.03% (0–0.32%) | 0. 27% (0-0.56%) |
| *Tonkinacris damingshanus* | 0. 03% (0–0.15%) | 0.03% (0–0.16%) | 0. 06% (0-**0.28**%) |
| *Tonkinacris meridionlis* | 0. 26% (0–0.30%) | 0.06% (0–0.32%) | 0 (0) |
| *Ognevia longipensis* | 0. 12% (0–0.30%) | 0.19% (0–**0.32**%) | 0. 11% (0-0.28%) |
| *Apalacris tonkinensis* | 0. 07% (0–0.15%) | 0.16% (0–**0.51**%) | 0. 32% (0-**0.60**%) |
| *Apalacris varicornis* | 1.23% (1.23%) | 0 (0) | **7.56**% (**7.56**%) |
| *Chondriacris rosea* | 0. 52% (0.15–0.92%) | 0.17% (0–0.34%) | 0. 38% (0-0.63%) |
| *Choroedocus capensis* | 0. 12% (0–0.30%) | 0.09% (0–0.17%) | 0.32% (0-**0.61**%) |
| *Xenocatantops brachycerus* | 0. 41% (0.30–0.61%) | 0 (0) | **5.04**% (0-**7.56**%) |
| *Oxya anagavisa* | 0. 08% (0–0.15%) | 0.22% (0–**0.53**%) | 0.40% (0-**0.89**%) |
| *Traulia angustipennis* | 0. 21% (0–0.46%) | 0. 11% (0–0.33%) | 0 (0) |
| *Gastrimargus marmoratus* | 0. 30% (0. 30%) | 0. 86% (0.37–**1.11**%) | 0 (0) |
| *Ceracris nigricornis* | 0. 40% (0–0.61%) | 0 (0) | 0.11% (0-0.29%) |
| *Phlaeoba antennata* | 0. 22% (0–0.46%) | 0.14% (0–0.37%) | 0 (0) |
| *Phlaeoba infumata* | n/c | n/c | n/c |
| *Ergatettix dorsiferus* | n/c | n/c | n/c |
| *Conocephalus longipennis* | 0. 46% (0. 46%) | 0 (0) | 0 (0) |
