# Supplementary material for: Molecular phylogeny and species delimitation of the genus Tonkinacris (Orthoptera, Acrididae, Melanoplinae) from China
Source: PLoS One. 2021 Apr 13;16(4):e0249431. doi: 10.1371/journal.pone.0249431 (PMC8043412; doi:10.1371/journal.pone.0249431)
Supplement: S5 Table — (DOCX) [file pone.0249431.s015.docx]

**S5 Table.** Mean genetic distances between species calculated from *COI* alignment.

|  | F_ton | L_mac | P_vit | E_mac | T_sin | T_dec | T_dam | T_mer | O_lon | A_ton | A_var | Chon_ros | Chor_cap | X_bra | Ox_ana | Tr_ang | G_mar | Ce_nig | Ph_ant | Ph_inf | Er_dor |
| --- | --- | --- | --- | --- | --- | --- | --- | --- | --- | --- | --- | --- | --- | --- | --- | --- | --- | --- | --- | --- | --- |
| L_mac | 0.0696 |  |  |  |  |  |  |  |  |  |  |  |  |  |  |  |  |  |  |  |  |
| P_vit | 0.0776 | 0.0729 |  |  |  |  |  |  |  |  |  |  |  |  |  |  |  |  |  |  |  |
| E_mac | 0.0806 | 0.0781 | 0.0479 |  |  |  |  |  |  |  |  |  |  |  |  |  |  |  |  |  |  |
| T_sin | 0.0911 | 0.0749 | 0.0757 | 0.0789 |  |  |  |  |  |  |  |  |  |  |  |  |  |  |  |  |  |
| T_dec | 0.0803 | 0.0774 | 0.0816 | 0.0866 | 0.0621 |  |  |  |  |  |  |  |  |  |  |  |  |  |  |  |  |
| T_dam | 0.0785 | 0.0729 | 0.0807 | 0.0885 | 0.0618 | 0.0102 |  |  |  |  |  |  |  |  |  |  |  |  |  |  |  |
| T_mer | 0.0882 | 0.0687 | 0.0782 | 0.0838 | 0.0570 | 0.0273 | 0.0298 |  |  |  |  |  |  |  |  |  |  |  |  |  |  |
| O_lon | 0.1070 | 0.1233 | 0.1181 | 0.1127 | 0.1243 | 0.1083 | 0.1063 | 0.1157 |  |  |  |  |  |  |  |  |  |  |  |  |  |
| A_ton | 0.1875 | 0.1842 | 0.1762 | 0.1696 | 0.1906 | 0.1816 | 0.1851 | 0.1751 | 0.1891 |  |  |  |  |  |  |  |  |  |  |  |  |
| A_var | 0.1804 | 0.1923 | 0.1798 | 0.1835 | 0.1997 | 0.1767 | 0.1812 | 0.1907 | 0.2097 | 0.1657 |  |  |  |  |  |  |  |  |  |  |  |
| C_ros | 0.1499 | 0.1269 | 0.1400 | 0.1380 | 0.1337 | 0.1274 | 0.1239 | 0.1347 | 0.1732 | 0.2006 | 0.1973 |  |  |  |  |  |  |  |  |  |  |
| C_cap | 0.1654 | 0.1559 | 0.1572 | 0.1486 | 0.1584 | 0.1496 | 0.1439 | 0.1416 | 0.1818 | 0.2066 | 0.2237 | 0.1630 |  |  |  |  |  |  |  |  |  |
| X_bra | 0.1466 | 0.1406 | 0.1304 | 0.1294 | 0.1485 | 0.1423 | 0.1455 | 0.1425 | 0.1680 | 0.2010 | 0.1990 | 0.1463 | 0.1834 |  |  |  |  |  |  |  |  |
| Ox_ana | 0.1429 | 0.1417 | 0.1445 | 0.1411 | 0.1656 | 0.1454 | 0.1442 | 0.1444 | 0.1595 | 0.1789 | 0.2033 | 0.1342 | 0.1575 | 0.1499 |  |  |  |  |  |  |  |
| Tr_ang | 0.1379 | 0.1414 | 0.1392 | 0.1467 | 0.1477 | 0.1396 | 0.1381 | 0.1351 | 0.1409 | 0.1998 | 0.1796 | 0.1680 | 0.1685 | 0.1690 | 0.1610 |  |  |  |  |  |  |
| G_mar | 0.1869 | 0.1772 | 0.1707 | 0.1682 | 0.1805 | 0.1850 | 0.1807 | 0.1801 | 0.2045 | 0.2092 | 0.2287 | 0.1950 | 0.1736 | 0.1924 | 0.1617 | 0.1834 |  |  |  |  |  |
| Ce_nig | 0.1651 | 0.1438 | 0.1539 | 0.1492 | 0.1550 | 0.1585 | 0.1536 | 0.1559 | 0.1763 | 0.1980 | 0.1993 | 0.1483 | 0.1881 | 0.1606 | 0.1613 | 0.1453 | 0.1942 |  |  |  |  |
| Ph_ant | 0.1665 | 0.1501 | 0.1493 | 0.1406 | 0.1577 | 0.1595 | 0.1547 | 0.1536 | 0.1727 | 0.2092 | 0.2129 | 0.1600 | 0.1853 | 0.1834 | 0.1544 | 0.1782 | 0.1717 | 0.1562 |  |  |  |
| Ph_inf | 0.1549 | 0.1460 | 0.1452 | 0.1451 | 0.1603 | 0.1490 | 0.1473 | 0.1473 | 0.1662 | 0.2099 | 0.1998 | 0.1633 | 0.1475 | 0.1549 | 0.1484 | 0.1560 | 0.1701 | 0.1331 | 0.1519 |  |  |
| Er_dor | 0.3176 | 0.3098 | 0.3045 | 0.3171 | 0.2894 | 0.2887 | 0.2837 | 0.2980 | 0.2930 | 0.3002 | 0.3082 | 0.2987 | 0.3228 | 0.3227 | 0.3160 | 0.3158 | 0.2846 | 0.2948 | 0.2898 | 0.3107 |  |
| Co_lon | 0.2751 | 0.2823 | 0.2768 | 0.2825 | 0.2678 | 0.2652 | 0.2607 | 0.2740 | 0.2852 | 0.2741 | 0.2909 | 0.2620 | 0.2975 | 0.2839 | 0.2842 | 0.2649 | 0.2567 | 0.2747 | 0.2879 | 0.2942 | 0.3239 |

Note. F_ton: *Fruhstorferiola tonkinensis*; L_mac: *Longgenacris maculacarina*; P_vit: *Paratonkinacris vittifemoralis*; Em_mac: *Emeiacris maculata*; T_sin: *Tonkinacris sinensis*; T_dec : *Tonkinacris decoratus*; T_dam: *Tonkinacris damingshanus*; T_mer: *Tonkinacris meridionlis*; O_lon: *Ognevia longipennis*; A_ton: *Apalacris tonkinensis*; A_var: *Apalacris varicornis*; Chon_ros: *Chondriacris rosea*; Chor_cap: *Choroedocus capensis*; X_bra: *Xenocatantops brachycerus*; Ox_ana: *Oxya anagavisa*; Tr_ang: *Traulia angustipennis*; G_mar: *Gastrimargus marmoratus*; Ce_nig: *Ceracris nigricornis*; Ph_ant: *Phlaeoba antennata*; Ph_inf: *Phlaeoba infumata*; Er_dor: *Ergatettix dorsiferus*; C_lon: *Conocephalus longipennis*.
