# Supplementary material for: Molecular phylogeny and species delimitation of the genus Tonkinacris (Orthoptera, Acrididae, Melanoplinae) from China
Source: PLoS One. 2021 Apr 13;16(4):e0249431. doi: 10.1371/journal.pone.0249431 (PMC8043412; doi:10.1371/journal.pone.0249431)
Supplement: S6 Table — (DOCX) [file pone.0249431.s016.docx]

**S6 Table.** Mean genetic distances between species calculated from ITS1 alignment.

|  | F_ton | L_mac | P_vit | E_mac | T_sin | T_dec | T_dam | T_mer | O_lon | A_ton | A_var | Chon_ros | Chor_cap | X_bra | Ox_ana | Tr_ang | G_mar | Ce_nig | Ph_ant | Ph_inf | Er_dor |
| --- | --- | --- | --- | --- | --- | --- | --- | --- | --- | --- | --- | --- | --- | --- | --- | --- | --- | --- | --- | --- | --- |
| L_mac | 0.032 |  |  |  |  |  |  |  |  |  |  |  |  |  |  |  |  |  |  |  |  |
| P_vit | 0.030 | 0.023 |  |  |  |  |  |  |  |  |  |  |  |  |  |  |  |  |  |  |  |
| E_mac | 0.030 | 0.027 | 0.002 |  |  |  |  |  |  |  |  |  |  |  |  |  |  |  |  |  |  |
| T_sin | 0.028 | 0.019 | 0.017 | 0.021 |  |  |  |  |  |  |  |  |  |  |  |  |  |  |  |  |  |
| T_dec | 0.029 | 0.022 | 0.020 | 0.024 | 0.004 |  |  |  |  |  |  |  |  |  |  |  |  |  |  |  |  |
| T_dam | 0.027 | 0.022 | 0.018 | 0.022 | 0.004 | 0.004 |  |  |  |  |  |  |  |  |  |  |  |  |  |  |  |
| T_mer | 0.029 | 0.022 | 0.020 | 0.024 | 0.004 | 0.000 | 0.004 |  |  |  |  |  |  |  |  |  |  |  |  |  |  |
| O_lon | 0.041 | 0.040 | 0.028 | 0.031 | 0.031 | 0.034 | 0.032 | 0.034 |  |  |  |  |  |  |  |  |  |  |  |  |  |
| A_ton | 0.136 | 0.141 | 0.122 | 0.124 | 0.130 | 0.129 | 0.129 | 0.127 | 0.136 |  |  |  |  |  |  |  |  |  |  |  |  |
| A_var | 0.123 | 0.129 | 0.120 | 0.120 | 0.120 | 0.119 | 0.116 | 0.119 | 0.121 | 0.071 |  |  |  |  |  |  |  |  |  |  |  |
| C_ros | 0.129 | 0.120 | 0.131 | 0.132 | 0.129 | 0.134 | 0.130 | 0.132 | 0.135 | 0.118 | 0.128 |  |  |  |  |  |  |  |  |  |  |
| C_cap | 0.128 | 0.133 | 0.129 | 0.129 | 0.127 | 0.128 | 0.128 | 0.126 | 0.137 | 0.119 | 0.113 | 0.155 |  |  |  |  |  |  |  |  |  |
| X_bra | 0.133 | 0.152 | 0.134 | 0.134 | 0.137 | 0.134 | 0.134 | 0.134 | 0.135 | 0.130 | 0.064 | 0.141 | 0.158 |  |  |  |  |  |  |  |  |
| Ox_ana | 0.139 | 0.147 | 0.132 | 0.133 | 0.141 | 0.142 | 0.141 | 0.141 | 0.135 | 0.149 | 0.100 | 0.154 | 0.153 | 0.157 |  |  |  |  |  |  |  |
| Tr_ang | 0.137 | 0.150 | 0.146 | 0.149 | 0.141 | 0.146 | 0.142 | 0.145 | 0.146 | 0.117 | 0.106 | 0.135 | 0.139 | 0.149 | 0.156 |  |  |  |  |  |  |
| G_mar | 0.132 | 0.143 | 0.134 | 0.133 | 0.138 | 0.139 | 0.136 | 0.137 | 0.132 | 0.101 | 0.089 | 0.117 | 0.126 | 0.115 | 0.121 | 0.114 |  |  |  |  |  |
| Ce_nig | 0.125 | 0.127 | 0.117 | 0.118 | 0.121 | 0.121 | 0.121 | 0.121 | 0.122 | 0.119 | 0.078 | 0.124 | 0.119 | 0.103 | 0.092 | 0.105 | 0.080 |  |  |  |  |
| Ph_ant | 0.139 | 0.129 | 0.125 | 0.126 | 0.132 | 0.127 | 0.129 | 0.128 | 0.125 | 0.112 | 0.091 | 0.117 | 0.130 | 0.115 | 0.149 | 0.105 | 0.101 | 0.091 |  |  |  |
| Ph_inf | 0.129 | 0.132 | 0.120 | 0.121 | 0.122 | 0.121 | 0.119 | 0.120 | 0.130 | 0.103 | 0.089 | 0.136 | 0.115 | 0.101 | 0.115 | 0.107 | 0.098 | 0.056 | 0.087 |  |  |
| Er_dor | 0.468 | 0.468 | 0.447 | 0.448 | 0.454 | 0.456 | 0.452 | 0.457 | 0.469 | 0.466 | 0.411 | 0.453 | 0.458 | 0.435 | 0.446 | 0.447 | 0.449 | 0.405 | 0.445 | 0.431 |  |
| Co_lon | 0.500 | 0.499 | 0.485 | 0.489 | 0.477 | 0.478 | 0.473 | 0.477 | 0.481 | 0.493 | 0.455 | 0.480 | 0.499 | 0.501 | 0.490 | 0.511 | 0.467 | 0.434 | 0.480 | 0.453 | 0.541 |

Note. F_ton: *Fruhstorferiola tonkinensis*; L_mac: *Longgenacris maculacarina*; P_vit: *Paratonkinacris vittifemoralis*; Em_mac: *Emeiacris maculata*; T_sin: *Tonkinacris sinensis*; T_dec : *Tonkinacris decoratus*; T_dam: *Tonkinacris damingshanus*; T_mer: *Tonkinacris meridionlis*; O_lon: *Ognevia longipennis*; A_ton: *Apalacris tonkinensis*; A_var: *Apalacris varicornis*; Chon_ros: *Chondriacris rosea*; Chor_cap: *Choroedocus capensis*; X_bra: *Xenocatantops brachycerus*; Ox_ana: *Oxya anagavisa*; Tr_ang: *Traulia angustipennis*; G_mar: *Gastrimargus marmoratus*; Ce_nig: *Ceracris nigricornis*; Ph_ant: *Phlaeoba antennata*; Ph_inf: *Phlaeoba infumata*; Er_dor: *Ergatettix dorsiferus*; C_lon: *Conocephalus longipennis*.
