# Supplementary material for: Molecular phylogeny and species delimitation of the genus Tonkinacris (Orthoptera, Acrididae, Melanoplinae) from China
Source: PLoS One. 2021 Apr 13;16(4):e0249431. doi: 10.1371/journal.pone.0249431 (PMC8043412; doi:10.1371/journal.pone.0249431)
Supplement: S7 Table — (DOCX) [file pone.0249431.s017.docx]

**S7 Table.** Mean genetic distances between species calculated from ITS2 alignment.

|  | F_ton | L_mac | P_vit | E_mac | T_sin | T_dec | T_dam | T_mer | O_lon | A_ton | A_var | Chon_ros | Chor_cap | X_bra | Ox_ana | Tr_ang | G_mar | Ce_nig | Ph_ant | Ph_inf | Er_dor |
| --- | --- | --- | --- | --- | --- | --- | --- | --- | --- | --- | --- | --- | --- | --- | --- | --- | --- | --- | --- | --- | --- |
| L_mac | 0.018 |  |  |  |  |  |  |  |  |  |  |  |  |  |  |  |  |  |  |  |  |
| P_vit | 0.019 | 0.018 |  |  |  |  |  |  |  |  |  |  |  |  |  |  |  |  |  |  |  |
| E_mac | 0.018 | 0.017 | 0.003 |  |  |  |  |  |  |  |  |  |  |  |  |  |  |  |  |  |  |
| T_sin | 0.012 | 0.012 | 0.012 | 0.011 |  |  |  |  |  |  |  |  |  |  |  |  |  |  |  |  |  |
| T_dec | 0.012 | 0.011 | 0.008 | 0.008 | 0.005 |  |  |  |  |  |  |  |  |  |  |  |  |  |  |  |  |
| T_dam | 0.009 | 0.010 | 0.011 | 0.010 | 0.004 | 0.004 |  |  |  |  |  |  |  |  |  |  |  |  |  |  |  |
| T_mer | 0.008 | 0.010 | 0.011 | 0.009 | 0.004 | 0.004 | 0.000 |  |  |  |  |  |  |  |  | . |  |  |  |  |  |
| O_lon | 0.018 | 0.021 | 0.016 | 0.017 | 0.015 | 0.011 | 0.015 | 0.015 |  |  |  |  |  |  |  |  |  |  |  |  |  |
| A_ton | 0.117 | 0.124 | 0.122 | 0.121 | 0.115 | 0.118 | 0.117 | 0.117 | 0.123 |  |  |  |  |  |  |  |  |  |  |  |  |
| A_var | 0.096 | 0.107 | 0.106 | 0.104 | 0.098 | 0.101 | 0.097 | 0.096 | 0.109 | 0.044 |  |  |  |  |  |  |  |  |  |  |  |
| C_ros | 0.129 | 0.136 | 0.139 | 0.138 | 0.133 | 0.135 | 0.136 | 0.136 | 0.139 | 0.108 | 0.100 |  |  |  |  |  |  |  |  |  |  |
| C_cap | 0.106 | 0.117 | 0.115 | 0.114 | 0.108 | 0.111 | 0.110 | 0.110 | 0.114 | 0.086 | 0.091 | 0.122 |  |  |  |  |  |  |  |  |  |
| X_bra | 0.090 | 0.100 | 0.099 | 0.097 | 0.091 | 0.094 | 0.090 | 0.090 | 0.103 | 0.056 | 0.038 | 0.097 | 0.094 |  |  |  |  |  |  |  |  |
| Ox_ana | 0.120 | 0.127 | 0.126 | 0.125 | 0.119 | 0.122 | 0.120 | 0.120 | 0.128 | 0.055 | 0.075 | 0.131 | 0.108 | 0.082 |  |  |  |  |  |  |  |
| Tr_ang | 0.095 | 0.104 | 0.108 | 0.106 | 0.100 | 0.103 | 0.099 | 0.098 | 0.110 | 0.097 | 0.096 | 0.120 | 0.089 | 0.095 | 0.119 |  |  |  |  |  |  |
| G_mar | 0.112 | 0.110 | 0.106 | 0.107 | 0.109 | 0.104 | 0.109 | 0.108 | 0.114 | 0.111 | 0.107 | 0.110 | 0.137 | 0.107 | 0.113 | 0.094 |  |  |  |  |  |
| Ce_nig | 0.149 | 0.155 | 0.159 | 0.158 | 0.155 | 0.158 | 0.153 | 0.153 | 0.165 | 0.101 | 0.092 | 0.133 | 0.119 | 0.091 | 0.127 | 0.123 | 0.135 |  |  |  |  |
| Ph_ant | 0.134 | 0.147 | 0.144 | 0.142 | 0.140 | 0.142 | 0.138 | 0.138 | 0.149 | 0.089 | 0.084 | 0.129 | 0.116 | 0.087 | 0.104 | 0.111 | 0.116 | 0.052 |  |  |  |
| Ph_inf | 0.124 | 0.133 | 0.134 | 0.133 | 0.130 | 0.133 | 0.128 | 0.128 | 0.139 | 0.089 | 0.081 | 0.114 | 0.106 | 0.083 | 0.089 | 0.093 | 0.103 | 0.067 | 0.043 |  |  |
| Er_dor | 0.580 | 0.577 | 0.571 | 0.569 | 0.567 | 0.576 | 0.566 | 0.567 | 0.603 | 0.561 | 0.541 | 0.525 | 0.550 | 0.535 | 0.549 | 0.499 | 0.596 | 0.546 | 0.554 | 0.563 |  |
| Co_lon | 0.642 | 0.658 | 0.641 | 0.641 | 0.649 | 0.643 | 0.644 | 0.643 | 0.644 | 0.582 | 0.582 | 0.606 | 0.548 | 0.576 | 0.606 | 0.530 | 0.634 | 0.664 | 0.626 | 0.653 | 0.798 |

Note. F_ton: *Fruhstorferiola tonkinensis*; L_mac: *Longgenacris maculacarina*; P_vit: *Paratonkinacris vittifemoralis*; Em_mac: *Emeiacris maculata*; T_sin: *Tonkinacris sinensis*; T_dec : *Tonkinacris decoratus*; T_dam: *Tonkinacris damingshanus*; T_mer: *Tonkinacris meridionlis*; O_lon: *Ognevia longipennis*; A_ton: *Apalacris tonkinensis*; A_var: *Apalacris varicornis*; Chon_ros: *Chondriacris rosea*; Chor_cap: *Choroedocus capensis*; X_bra: *Xenocatantops brachycerus*; Ox_ana: *Oxya anagavisa*; Tr_ang: *Traulia angustipennis*; G_mar: *Gastrimargus marmoratus*; Ce_nig: *Ceracris nigricornis*; Ph_ant: *Phlaeoba antennata*; Ph_inf: *Phlaeoba infumata*; Er_dor: *Ergatettix dorsiferus*; C_lon: *Conocephalus longipennis*.

**Table S8. Haplotyptes of COI detected from samples of *Tonkinacris spp*.**

| Haplotype number | Individuals involved | Haplotype number | Individuals involved |
| --- | --- | --- | --- |
| 1 | ***Tonkinacris sinensis***  **Gaozhai, Guangxi:** gh020–024, gh108, gh110, gh119, gh122,  **Gaoji, Guangxi:** gh035, gh036, gh038, gh039.  **Diding, Guangxi:** gh031, gh032, gh033, gh034.  **Longshi, Nonggang, Guangxi:** gh134, gh137. | 10 | ***Tonkinacris sinensis***  **Damingshan, Guangxi:** gh098, gh100. |
| 2 | ***Tonkinacris sinensis***  **Gaozhai, Guangxi:** gh025, gh028, gh029. | 11 | ***Tonkinacris sinensis***  **Emeishan, Sichuan:** gh103, gh105, gh106, gh107.  **Longshi, Nonggang, Guangxi:** gh136. |
| 3 | ***Tonkinacris sinensis***  **Gaozhai, Guangxi:** gh026, gh027.  **Diding, Guangxi:** gh030. | 12 | ***Tonkinacris sinensis***  **Emeishan, Sichuan:** gh104. |
| 4 | ***Tonkinacris sinensis***  **Gaozhai, Guangxi:** gh109, gh111, gh120, gh121. | 13 | ***Tonkinacris sinensis***  **Longshi, Nonggang, Guangxi:** gh133. |
| 5 | ***Tonkinacris sinensis***  **Gaozhai, Guangxi:** gh112. | 14 | ***Tonkinacris sinensis***  **Longshi, Nonggang, Guangxi:** gh135. |
| 6 | ***Tonkinacris sinensis***  **Gaozhai, Guangxi:** gh118  **Dayaoshan, Guangxi:** gh093, gh094, gh097.  **Damingshan, Guangxi:** gh102. | 15 | ***Tonkinacris sinensis***  **Longshi, Nonggang, Guangxi:** gh138. |
| 7 | ***Tonkinacris sinensis***  **Gaoji, Guangxi:** gh037. | 16 | ***Tonkinacris sinensis***  **Yong'an, Guangxi:** gl0257, gl0260. |
| 8 | ***Tonkinacris sinensis***  **Dayaoshan, Guangxi:** gh095.  **Damingshan, Guangxi:** gh099, gh101. | 17 | ***Tonkinacris sinensis***  **Yong'an, Guangxi:** gl0258, gl0259, gl0261. |
| 9 | ***Tonkinacris sinensis***  **Dayaoshan, Guangxi:** gh096. | 18 | ***Tonkinacris decoratus***  **Longfang, Nonggang, Guangxi:** gh050*, gh053, gh054*.  **Longjiang, Nonggang, Guangxi:** gh062*. |
| 19 | ***Tonkinacris decoratus***  **Longfang, Nonggang, Guangxi:** gh051. | 25 | ***Tonkinacris meridionalis***  **Longrui, Guangxi:** gh227, gh228. |
| 20 | ***Tonkinacris decoratus***  **Longfang, Nonggang, Guangxi:** gh052, gh069*.  **Longjiang, Nonggang, Guangxi:** gh060, gh061, gh063*, gh064.  **Longshi, Nonggang, Guangxi:** gh139, gh140, gh141, gh142, gh143 | 26 | ***Tonkinacris meridionalis***  **Longrui, Guangxi:** gh229. |
| 21 | ***Tonkinacris decoratus***  **Longfang, Nonggang, Guangxi:** gh065, gh068*. | 27 | ***Tonkinacris meridionalis***  **Longrui, Guangxi:** gh230. |
| 22 | ***Tonkinacris decoratus***  **Longfang, Nonggang, Guangxi:** gh066, gh067. | 28 | ***Tonkinacris meridionalis***  **Longrui, Guangxi:** gh231. |
| 23 | ***Tonkinacris damingshanus***  **Damingshan, Guangxi:** gh128–132, 149–151, 153. | 29 | ***Tonkinacris meridionalis***  **Longrui, Guangxi:** gh232–234, 236. |
| 24 | ***Tonkinacris damingshanus***  **Damingshan, Guangxi:** gh152. | 30 | ***Tonkinacris meridionalis***  **Longrui, Guangxi:** gh235. |

**Note.** The asterisk (*) indicates the individuals of *T. decoratus* with distinct or indistinct black transverse maculation on the base of the upper surface of hind femur.

**Table S9. Haplotyptes of ITS1 detected from samples of *Tonkinacris spp*.**

| Haplotype number | Individuals involved | Haplotype number | Individuals involved |
| --- | --- | --- | --- |
| 1 | ***Tonkinacris sinensis*:** gh020, gh022. | 10 | ***Tonkinacris sinensis*:** gh103, gh105, gh106. |
| 2 | ***Tonkinacris sinensis*:** gh021, gh023–027, gh029, gh108–112, gh118–122, gh030, gh031, gh034, gh037, gh093–098, gh100, gh101, gh133–138, gl0257–0261. | 11 | ***Tonkinacris sinensis*:** gh104. |
| 3 | ***Tonkinacris sinensis*:** gh028. | 12 | ***Tonkinacris decoratus:*** gh050–054, gh061–063, gh065–069, gh139–143. |
| 4 | ***Tonkinacris sinensis*:** gh032. | 13 | ***Tonkinacris decoratus:*** gh060. |
| 5 | ***Tonkinacris sinensis*:** gh033, gh038, gh039. | 14 | ***Tonkinacris decoratus:*** gh064. |
| 6 | ***Tonkinacris sinensis*:** gh035. | 15 | ***Tonkinacris damingshanus*:** gh128, gh129, gh131, gh132, gh149–153 |
| 7 | ***Tonkinacris sinensis*:** gh036. | 16 | ***Tonkinacris damingshanus*:** gh130. |
| 8 | ***Tonkinacris sinensis*:** gh099. | 17 | ***Tonkinacris meridionalis*:** gh227–235. |
| 9 | ***Tonkinacris sinensis*:** gh102. | 18 | ***Tonkinacris meridionalis*:** gh236. |

**Table S10. Haplotyptes of ITS2 detected from samples of *Tonkinacris spp*.**

| Haplotype number | Individuals involved | Haplotype number | Individuals involved |
| --- | --- | --- | --- |
| 1 | ***Tonkinacris sinensis*:** gh020, gh024, gh108–112, gh118, gh119, gh121, gh122, gh093–097, gh099–102, gh133, gh134, gh137, gl0257, gl0259, gl0261. | 5 | ***Tonkinacris sinensis*:** gh027. |
| 2 | ***Tonkinacris sinensis*:** gh021, gh037. | 6 | ***Tonkinacris sinensis*:** gh120. |
| 3 | ***Tonkinacris sinensis*:** gh022, gh023, gh031–036, gh038, gh039. | 7 | ***Tonkinacris sinensis*:** gh098, gh103–107, gh135, gh136, gh138, gl0258, gl0260.  ***Tonkinacris decoratus:* gh062, gh064, gh139**–143  ***Tonkinacris damingshanus*:** gh128–132, gh149–151, gh153.  ***Tonkinacris meridionalis*:** gh227–236. |
| 4 | ***Tonkinacris sinensis*:** gh025, gh026, gh028–030,  ***Tonkinacris decoratus:*** gh050–054, gh060, gh061, gh063, gh065–069. | 8 | ***Tonkinacris damingshanus*:** gh152. |

**Table S11. Putative species delineated from COI alignment using GMYC model.**

| GMYC species | Morphospecies/Sample name/locality |
| --- | --- |
| 1 | ***Choroedocus capensis:***  gh170-173 / Longjiang, Nonggang, Longzhou, Guangxi.  gh184-191 / Sanlidian, Guilin, Guangxi. |
| 2 | ***Gastrimargus_marmoratus*:** gh181-183 / Longjiang, Nonggang, Longzhou, Guangxi. |
| 3 | ***Traulia angustipennis*:** gh221-226 / Sanlian, Nonggang, Longzhouy, Guangxi |
| 4 | ***Ognevia longipennis*:** gl0252-0256 / Yangjiaping, Zhuolu, Hebei. |
| 5 | ***Tonkinacris sinensis*:**  gh093-097 / Qigongli, Dayaoshan, Jinxiu, Guangxi.  gh099, gh101, gh102 / Xiashuiyuan, Damingshan, Shanglin, Guangxi.  gh118 / Gaozhai, Xing'an, Guangxi. |
| 6 | ***Tonkinacris sinensis*:**  gl0257-0261 / Yong'an, Xing'an, Guangxi.  gh020-024, gh108-111, gh119-122 / Gaozhai, Xing'an, Guangxi.  gh031-034 / Diding, Jingxi, Guangxi.  gh035-039 / Gaoji Town, Sanjiang, Guangxi.  gh098, gh100 / Xiashuiyuan, Damingshan, Shanglin, Guangxi.  gh103-107 / Fuhusi, Emeishan, Leshan County, Sichuan.  gh133, gh134, gh136, gh137 / Longshi, Nonggang, Longzhou, Guangxi. |
| 7 | ***Tonkinacris sinensis*:**  gh025-029 / Gaozhai, Xing'an, Guangxi.  gh030 / Diding, Jingxi, Guangxi.  gh135, gh138 / Longshi, Nonggang, Longzhou, Guangxi. |
| 8 | ***Tonkinacris sinensis*:** gh112 / Gaozhai, Xing'an, Guangxi. |
| 9 | ***Tonkinacris meridionalis:*** Longrui, Longzhou, Guangxi. |
| 10 | ***Tonkinacris_decoratus:***  gh050-054, 065-069 / Longfang, Nonggang, Longzhou, Guangxi.  gh060-064 / Longjiang, Nonggang, Longzhou, Guangxi.  gh139-143 / Longshi, Nonggang, Longzhou, Guangxi.  ***Tonkinacris damingshanus*:**  gh128-132, gh149-153 /Yuanshisenlin, Damingshan, Wuming, Guangxi. |
| 11 | ***Emeiacris maculata*:** gl0241-0246/Hengshan, Hunan. |
| 12 | ***Emeiacris maculata*:** gh075-079, gh088-092/Emeishan, Sichuan. |
| 13 | ***Paratonkinacris vittifemoralis*:** gl0251/Gaozhai, Guangxi. |
| 14 | ***Paratonkinacris vittifemoralis*:** gh045-049, gl0247-0250/Gaozhai, Guangxi. |
| 15 | ***Longgenacris_maculacarina*:**  **g**h015-019, gh144-148, gh159-163 / Nonggang, Longzhou, Guangxi. |
| 16 | ***Fruhstorferiola tonkinensis*:** gl0089-0094/Yong'an, Guangxi. |
| 17 | ***Oxya anagavisa*:** gh070-074 / Damingshan, Wuming, Guangxi.  gh179-180, gh202-206/ Nonggang, Longzhou, Guangxi. |
| 18 | ***Xenocatantops brachycerus*:** gh192-194 / Nonggang, Longzhou, Guangxi. |
| 19 | ***Chondracris rosea:*** gh174-178 / Nonggang, Longzhou, Guangxi. |
| 20 | ***Ceracris_nigricornis*:** gh197-201 / Nonggang, Longzhou, Guangxi. |
| 21 | ***Phlaeoba antennata*:** gh212-214, gh216-220 / Nonggang, Longzhou, Guangxi. |
| 22 | ***Phlaeoba infumata*:** gh215/ Nonggang, Longzhou, Guangxi. |
| 23 | ***Apalacris varicornis*:** gh195-196 / Nonggang, Longzhou, Guangxi. |
| 24 | ***Apalacris tonkinensis*:** gh164-168, gh207-211 / Nonggang, Longzhou, Guangxi. |
| 25 | ***Ergatettix dorsiferus*:** gh247/Fangchenggang, Guangxi Province |
| 26 | ***Conocephalus longipennis*:** gh242-243 / Guilin, Guangxi. |
