# Supplementary material for: Molecular phylogeny and species delimitation of the genus Tonkinacris (Orthoptera, Acrididae, Melanoplinae) from China
Source: PLoS One. 2021 Apr 13;16(4):e0249431. doi: 10.1371/journal.pone.0249431 (PMC8043412; doi:10.1371/journal.pone.0249431)
Supplement: S8 Table — (DOCX) [file pone.0249431.s018.docx]

**S8 Table.** Haplotyptes of COI detected from samples of *Tonkinacris spp*.

| Haplotype number | Individuals involved | Haplotype number | Individuals involved |
| --- | --- | --- | --- |
| 1 | ***Tonkinacris sinensis***  **Gaozhai, Guangxi:** gh020–024, gh108, gh110, gh119, gh122,  **Gaoji, Guangxi:** gh035, gh036, gh038, gh039.  **Diding, Guangxi:** gh031, gh032, gh033, gh034.  **Longshi, Nonggang, Guangxi:** gh134, gh137. | 10 | ***Tonkinacris sinensis***  **Damingshan, Guangxi:** gh098, gh100. |
| 2 | ***Tonkinacris sinensis***  **Gaozhai, Guangxi:** gh025, gh028, gh029. | 11 | ***Tonkinacris sinensis***  **Emeishan, Sichuan:** gh103, gh105, gh106, gh107.  **Longshi, Nonggang, Guangxi:** gh136. |
| 3 | ***Tonkinacris sinensis***  **Gaozhai, Guangxi:** gh026, gh027.  **Diding, Guangxi:** gh030. | 12 | ***Tonkinacris sinensis***  **Emeishan, Sichuan:** gh104. |
| 4 | ***Tonkinacris sinensis***  **Gaozhai, Guangxi:** gh109, gh111, gh120, gh121. | 13 | ***Tonkinacris sinensis***  **Longshi, Nonggang, Guangxi:** gh133. |
| 5 | ***Tonkinacris sinensis***  **Gaozhai, Guangxi:** gh112. | 14 | ***Tonkinacris sinensis***  **Longshi, Nonggang, Guangxi:** gh135. |
| 6 | ***Tonkinacris sinensis***  **Gaozhai, Guangxi:** gh118  **Dayaoshan, Guangxi:** gh093, gh094, gh097.  **Damingshan, Guangxi:** gh102. | 15 | ***Tonkinacris sinensis***  **Longshi, Nonggang, Guangxi:** gh138. |
| 7 | ***Tonkinacris sinensis***  **Gaoji, Guangxi:** gh037. | 16 | ***Tonkinacris sinensis***  **Yong'an, Guangxi:** gl0257, gl0260. |
| 8 | ***Tonkinacris sinensis***  **Dayaoshan, Guangxi:** gh095.  **Damingshan, Guangxi:** gh099, gh101. | 17 | ***Tonkinacris sinensis***  **Yong'an, Guangxi:** gl0258, gl0259, gl0261. |
| 9 | ***Tonkinacris sinensis***  **Dayaoshan, Guangxi:** gh096. | 18 | ***Tonkinacris decoratus***  **Longfang, Nonggang, Guangxi:** gh050*, gh053, gh054*.  **Longjiang, Nonggang, Guangxi:** gh062*. |
| 19 | ***Tonkinacris decoratus***  **Longfang, Nonggang, Guangxi:** gh051. | 25 | ***Tonkinacris meridionalis***  **Longrui, Guangxi:** gh227, gh228. |
| 20 | ***Tonkinacris decoratus***  **Longfang, Nonggang, Guangxi:** gh052, gh069*.  **Longjiang, Nonggang, Guangxi:** gh060, gh061, gh063*, gh064.  **Longshi, Nonggang, Guangxi:** gh139, gh140, gh141, gh142, gh143 | 26 | ***Tonkinacris meridionalis***  **Longrui, Guangxi:** gh229. |
| 21 | ***Tonkinacris decoratus***  **Longfang, Nonggang, Guangxi:** gh065, gh068*. | 27 | ***Tonkinacris meridionalis***  **Longrui, Guangxi:** gh230. |
| 22 | ***Tonkinacris decoratus***  **Longfang, Nonggang, Guangxi:** gh066, gh067. | 28 | ***Tonkinacris meridionalis***  **Longrui, Guangxi:** gh231. |
| 23 | ***Tonkinacris damingshanus***  **Damingshan, Guangxi:** gh128–132, 149–151, 153. | 29 | ***Tonkinacris meridionalis***  **Longrui, Guangxi:** gh232–234, 236. |
| 24 | ***Tonkinacris damingshanus***  **Damingshan, Guangxi:** gh152. | 30 | ***Tonkinacris meridionalis***  **Longrui, Guangxi:** gh235. |

**Note.** The asterisk (*) indicates the individuals of *T. decoratus* with distinct or indistinct black transverse maculation on the base of the upper surface of hind femur.
