# Supplementary material for: Molecular phylogeny and species delimitation of the genus Tonkinacris (Orthoptera, Acrididae, Melanoplinae) from China
Source: PLoS One. 2021 Apr 13;16(4):e0249431. doi: 10.1371/journal.pone.0249431 (PMC8043412; doi:10.1371/journal.pone.0249431)
Supplement: S9 Table — (DOCX) [file pone.0249431.s019.docx]

**S9 Table. Haplotyptes of ITS1 detected from samples of *Tonkinacris spp*.**

| Haplotype number | Individuals involved | Haplotype number | Individuals involved |
| --- | --- | --- | --- |
| 1 | ***Tonkinacris sinensis*:** gh020, gh022. | 10 | ***Tonkinacris sinensis*:** gh103, gh105, gh106. |
| 2 | ***Tonkinacris sinensis*:** gh021, gh023–027, gh029, gh108–112, gh118–122, gh030, gh031, gh034, gh037, gh093–098, gh100, gh101, gh133–138, gl0257–0261. | 11 | ***Tonkinacris sinensis*:** gh104. |
| 3 | ***Tonkinacris sinensis*:** gh028. | 12 | ***Tonkinacris decoratus:*** gh050–054, gh061–063, gh065–069, gh139–143. |
| 4 | ***Tonkinacris sinensis*:** gh032. | 13 | ***Tonkinacris decoratus:*** gh060. |
| 5 | ***Tonkinacris sinensis*:** gh033, gh038, gh039. | 14 | ***Tonkinacris decoratus:*** gh064. |
| 6 | ***Tonkinacris sinensis*:** gh035. | 15 | ***Tonkinacris damingshanus*:** gh128, gh129, gh131, gh132, gh149–153 |
| 7 | ***Tonkinacris sinensis*:** gh036. | 16 | ***Tonkinacris damingshanus*:** gh130. |
| 8 | ***Tonkinacris sinensis*:** gh099. | 17 | ***Tonkinacris meridionalis*:** gh227–235. |
| 9 | ***Tonkinacris sinensis*:** gh102. | 18 | ***Tonkinacris meridionalis*:** gh236. |
