# Supplementary material for: Molecular phylogeny and species delimitation of the genus Tonkinacris (Orthoptera, Acrididae, Melanoplinae) from China
Source: PLoS One. 2021 Apr 13;16(4):e0249431. doi: 10.1371/journal.pone.0249431 (PMC8043412; doi:10.1371/journal.pone.0249431)
Supplement: S10 Table — (DOCX) [file pone.0249431.s020.docx]

**S10 Table. Haplotyptes of ITS2 detected from samples of *Tonkinacris spp*.**

| Haplotype number | Individuals involved | Haplotype number | Individuals involved |
| --- | --- | --- | --- |
| 1 | ***Tonkinacris sinensis*:** gh020, gh024, gh108–112, gh118, gh119, gh121, gh122, gh093–097, gh099–102, gh133, gh134, gh137, gl0257, gl0259, gl0261. | 5 | ***Tonkinacris sinensis*:** gh027. |
| 2 | ***Tonkinacris sinensis*:** gh021, gh037. | 6 | ***Tonkinacris sinensis*:** gh120. |
| 3 | ***Tonkinacris sinensis*:** gh022, gh023, gh031–036, gh038, gh039. | 7 | ***Tonkinacris sinensis*:** gh098, gh103–107, gh135, gh136, gh138, gl0258, gl0260.  ***Tonkinacris decoratus:* gh062, gh064, gh139**–143  ***Tonkinacris damingshanus*:** gh128–132, gh149–151, gh153.  ***Tonkinacris meridionalis*:** gh227–236. |
| 4 | ***Tonkinacris sinensis*:** gh025, gh026, gh028–030,  ***Tonkinacris decoratus:*** gh050–054, gh060, gh061, gh063, gh065–069. | 8 | ***Tonkinacris damingshanus*:** gh152. |
