# Supplementary material for: Molecular phylogeny and species delimitation of the genus Tonkinacris (Orthoptera, Acrididae, Melanoplinae) from China
Source: PLoS One. 2021 Apr 13;16(4):e0249431. doi: 10.1371/journal.pone.0249431 (PMC8043412; doi:10.1371/journal.pone.0249431)
Supplement: S11 Table — (DOCX) [file pone.0249431.s021.docx]

**S11 Table. Putative species delineated from COI alignment using GMYC model.**

| GMYC species | Morphospecies/Sample name/locality |
| --- | --- |
| 1 | ***Choroedocus capensis:***  gh170-173 / Longjiang, Nonggang, Longzhou, Guangxi.  gh184-191 / Sanlidian, Guilin, Guangxi. |
| 2 | ***Gastrimargus_marmoratus*:** gh181-183 / Longjiang, Nonggang, Longzhou, Guangxi. |
| 3 | ***Traulia angustipennis*:** gh221-226 / Sanlian, Nonggang, Longzhouy, Guangxi |
| 4 | ***Ognevia longipennis*:** gl0252-0256 / Yangjiaping, Zhuolu, Hebei. |
| 5 | ***Tonkinacris sinensis*:**  gh093-097 / Qigongli, Dayaoshan, Jinxiu, Guangxi.  gh099, gh101, gh102 / Xiashuiyuan, Damingshan, Shanglin, Guangxi.  gh118 / Gaozhai, Xing'an, Guangxi. |
| 6 | ***Tonkinacris sinensis*:**  gl0257-0261 / Yong'an, Xing'an, Guangxi.  gh020-024, gh108-111, gh119-122 / Gaozhai, Xing'an, Guangxi.  gh031-034 / Diding, Jingxi, Guangxi.  gh035-039 / Gaoji Town, Sanjiang, Guangxi.  gh098, gh100 / Xiashuiyuan, Damingshan, Shanglin, Guangxi.  gh103-107 / Fuhusi, Emeishan, Leshan County, Sichuan.  gh133, gh134, gh136, gh137 / Longshi, Nonggang, Longzhou, Guangxi. |
| 7 | ***Tonkinacris sinensis*:**  gh025-029 / Gaozhai, Xing'an, Guangxi.  gh030 / Diding, Jingxi, Guangxi.  gh135, gh138 / Longshi, Nonggang, Longzhou, Guangxi. |
| 8 | ***Tonkinacris sinensis*:** gh112 / Gaozhai, Xing'an, Guangxi. |
| 9 | ***Tonkinacris meridionalis:*** Longrui, Longzhou, Guangxi. |
| 10 | ***Tonkinacris_decoratus:***  gh050-054, 065-069 / Longfang, Nonggang, Longzhou, Guangxi.  gh060-064 / Longjiang, Nonggang, Longzhou, Guangxi.  gh139-143 / Longshi, Nonggang, Longzhou, Guangxi.  ***Tonkinacris damingshanus*:**  gh128-132, gh149-153 /Yuanshisenlin, Damingshan, Wuming, Guangxi. |
| 11 | ***Emeiacris maculata*:** gl0241-0246/Hengshan, Hunan. |
| 12 | ***Emeiacris maculata*:** gh075-079, gh088-092/Emeishan, Sichuan. |
| 13 | ***Paratonkinacris vittifemoralis*:** gl0251/Gaozhai, Guangxi. |
| 14 | ***Paratonkinacris vittifemoralis*:** gh045-049, gl0247-0250/Gaozhai, Guangxi. |
| 15 | ***Longgenacris_maculacarina*:**  **g**h015-019, gh144-148, gh159-163 / Nonggang, Longzhou, Guangxi. |
| 16 | ***Fruhstorferiola tonkinensis*:** gl0089-0094/Yong'an, Guangxi. |
| 17 | ***Oxya anagavisa*:** gh070-074 / Damingshan, Wuming, Guangxi.  gh179-180, gh202-206/ Nonggang, Longzhou, Guangxi. |
| 18 | ***Xenocatantops brachycerus*:** gh192-194 / Nonggang, Longzhou, Guangxi. |
| 19 | ***Chondracris rosea:*** gh174-178 / Nonggang, Longzhou, Guangxi. |
| 20 | ***Ceracris_nigricornis*:** gh197-201 / Nonggang, Longzhou, Guangxi. |
| 21 | ***Phlaeoba antennata*:** gh212-214, gh216-220 / Nonggang, Longzhou, Guangxi. |
| 22 | ***Phlaeoba infumata*:** gh215/ Nonggang, Longzhou, Guangxi. |
| 23 | ***Apalacris varicornis*:** gh195-196 / Nonggang, Longzhou, Guangxi. |
| 24 | ***Apalacris tonkinensis*:** gh164-168, gh207-211 / Nonggang, Longzhou, Guangxi. |
| 25 | ***Ergatettix dorsiferus*:** gh247/Fangchenggang, Guangxi Province |
| 26 | ***Conocephalus longipennis*:** gh242-243 / Guilin, Guangxi. |
