# Supplementary material for: Molecular phylogeny and species delimitation of the genus Tonkinacris (Orthoptera, Acrididae, Melanoplinae) from China
Source: PLoS One. 2021 Apr 13;16(4):e0249431. doi: 10.1371/journal.pone.0249431 (PMC8043412; doi:10.1371/journal.pone.0249431)
Supplement: Attachment — Submitted filename: Response to reviewers.docx [file pone.0249431.s022.docx]

Dear editor,

We have fully revised the manuscript according to the comments of the reviewers. Below is our response to the reveiwers.

1. We have fully reviewed the reference list to ensure that it is complete and correct.

2. We have formatted the manuscript to meet PLOS ONE's style requirements according to the guidlines in the PLOS ONE style templates found at https://journals.plos.org/plosone/s/file?id=wjVg/PLOSOne_formatting_sample_main_body.pdf and [https://journals.plos.org/plosone/s/file?id=ba62/PLOSOne_formatting_sample_title_authors_affiliations.pdf](about:blank).

3. We have no amended statement on financial disclosure for the manuscript.

4. The ground map of figure 1 in the manuscript meets the requirement of publication in PLOSOne. There is no need to get a written permission from the copyright holder. The ground map of figure 1 was published by the Ministry of Natural Resources of the People's Republic of China with the Permission number GS(2019)1679 and is available freely at http://bzdt.ch.mnr.gov.cn/browse.html?picId=%224o28b0625501ad13015501ad2bfc0281%22. We have included these informations in the legend of figure 1, did not make any modification to the content of the ground map and just added the spepcies distribution information onto it. Any standard ground map published in the website "Standard Map Service" (http://bzdt.ch.mnr.gov.cn/) by the Ministry of Natural Resources of the People's Republic of China can be used freely by anyone as illustrations in propaganda, book, journal and newspaper, and ground images for advertisement and crafts design, only if he/she does not make any modification to any content of the original ground map and includes the permission number in the map. The statement for freely using those public standard maps can be found at http://bzdt.ch.mnr.gov.cn/ (see the screen shotsnapes below).

5. We have revised the main text according to the comments of the reviewers (see the manuscript with trace changes).

6. For the first comment of Reviewer #1: For the study of molecular phylogeny with the genus *Tonkinacris*, the sample from another suborder/family is totally superfluous. While the comment of the reviewer is right, the sampled taxon of outgroup from another suborder Ensifera has no negative influence on our result. We will fully consider this suggest in our future research works.

7. The reviewer #2 pointed out that all figures are not clear due to low photo resolution. But this is not the case. All of the figures uploeaded onto the system have enough resolutions, but what the reviewers see in the pdf draft of the manuscirpt are the ones reduced when creating pdf file. We have updated figures 3 and 4 with new files having higher resolutions.

8. We have invite native English speakers to help us polish the manuscript.
